# Innate immunity restricts *Citrobacter rodentium* A/E pathogenesis initiation to an early window of opportunity

**DOI:** 10.1371/journal.ppat.1006476

**Published:** 2017-06-29

**Authors:** Stefanie Buschor, Miguelangel Cuenca, Stephanie S. Uster, Olivier P. Schären, Maria L. Balmer, Miguel A. Terrazos, Christian M. Schürch, Siegfried Hapfelmeier

**Affiliations:** 1 Institute for Infectious Diseases, University of Bern, Bern, Switzerland; 2 Graduate School GCB, University of Bern, Bern, Switzerland; 3 Department of Biomedicine, Immunobiology, University of Basel, Basel, Switzerland; 4 Institute of Pathology, University of Bern, Bern, Switzerland; University of Toronto, CANADA

## Abstract

*Citrobacter rodentium* infection is a mouse model for the important human diarrheal infection caused by enteropathogenic *E*. *coli* (EPEC). The pathogenesis of both species is very similar and depends on their unique ability to form intimately epithelium-adherent microcolonies, also known as “attachment/effacement” (A/E) lesions. These microcolonies must be dynamic and able to self-renew by continuous re-infection of the rapidly regenerating epithelium. It is unknown whether sustained epithelial A/E lesion pathogenesis is achieved through re-infection by planktonic bacteria from the luminal compartment or local spread of sessile bacteria without a planktonic phase. Focusing on the earliest events as *C*. *rodentium* becomes established, we show here that all colonic epithelial A/E microcolonies are clonal bacterial populations, and thus depend on local clonal growth to persist. In wild-type mice, microcolonies are established exclusively within the first 18 hours of infection. These early events shape the ongoing intestinal geography and severity of infection despite the continuous presence of phenotypically virulent luminal bacteria. Mechanistically, induced resistance to A/E lesion de-novo formation is mediated by TLR-MyD88/Trif-dependent signaling and is induced specifically by virulent *C*. *rodentium* in a virulence gene-dependent manner. Our data demonstrate that the establishment phase of *C*. *rodentium* pathogenesis *in vivo* is restricted to a very short window of opportunity that determines both disease geography and severity.

## Author summary

The so-called “attaching and effacing” (A/E) pathogens enteropathogenic *E*. *coli* (EPEC) and enterohemorrhagic *E*. *coli* (EHEC) cause serious human diarrheal infections by adhering to and damaging the intestinal epithelium. Previous work on the mouse A/E pathogen *Citrobacter rodentium* has established that host adaptive immune response and intestinal microbiota cooperate to control the epithelial infection and colonization with this pathogen. We found that this is complemented by a rapid pathogen-induced mucosal innate immune response that is essential to prevent excessive pathogenesis before adaptive immunity takes effect. Its effectiveness is demonstrated by the fact that it normally limits the duration during which the bacteria can induce epithelial lesions to the first 18 hours of infection. Later, luminal virulent bacteria can no longer induce new A/E lesions, but those induced already in the first 18 hours of infection persist through localized epithelial re-infection. Severity of the disease at the peak of infection is consequently shaped by the early events of the first 18 hours. This information may be important for the development of effective therapies and vaccines.

## Introduction

Enteropathogenic *Escherichia coli* (EPEC) and enterohemorrhagic *E*. *coli* (EHEC) serotype O157:H7 remain important causes of human diarrhea and mortality worldwide [1,2]. *Citrobacter rodentium* is a natural pathogen of mice which is used to model human EPEC and EHEC infection, as it shares the pathogenic mechanism of epithelial “attachment and effacement” (A/E). These pathogens attach to and colonize the intestinal mucosal surface in the form of epithelium-adherent microcolonies, also known as A/E lesions [3], comprised of bacteria that intimately attach to the apical surface of epithelial cells and induce the local destruction (“effacement”) of the epithelial brush border. This triggers reactive colonic epithelial hyperplasia, inflammation and the induction of IL-17-producing CD4 helper T (Th17) cells and IL-22-producing type 3 innate lymphoid cells (ILC3) [4,5]. *C*. *rodentium* has thus become a valued A/E infection mouse model.

**Fig 1:**
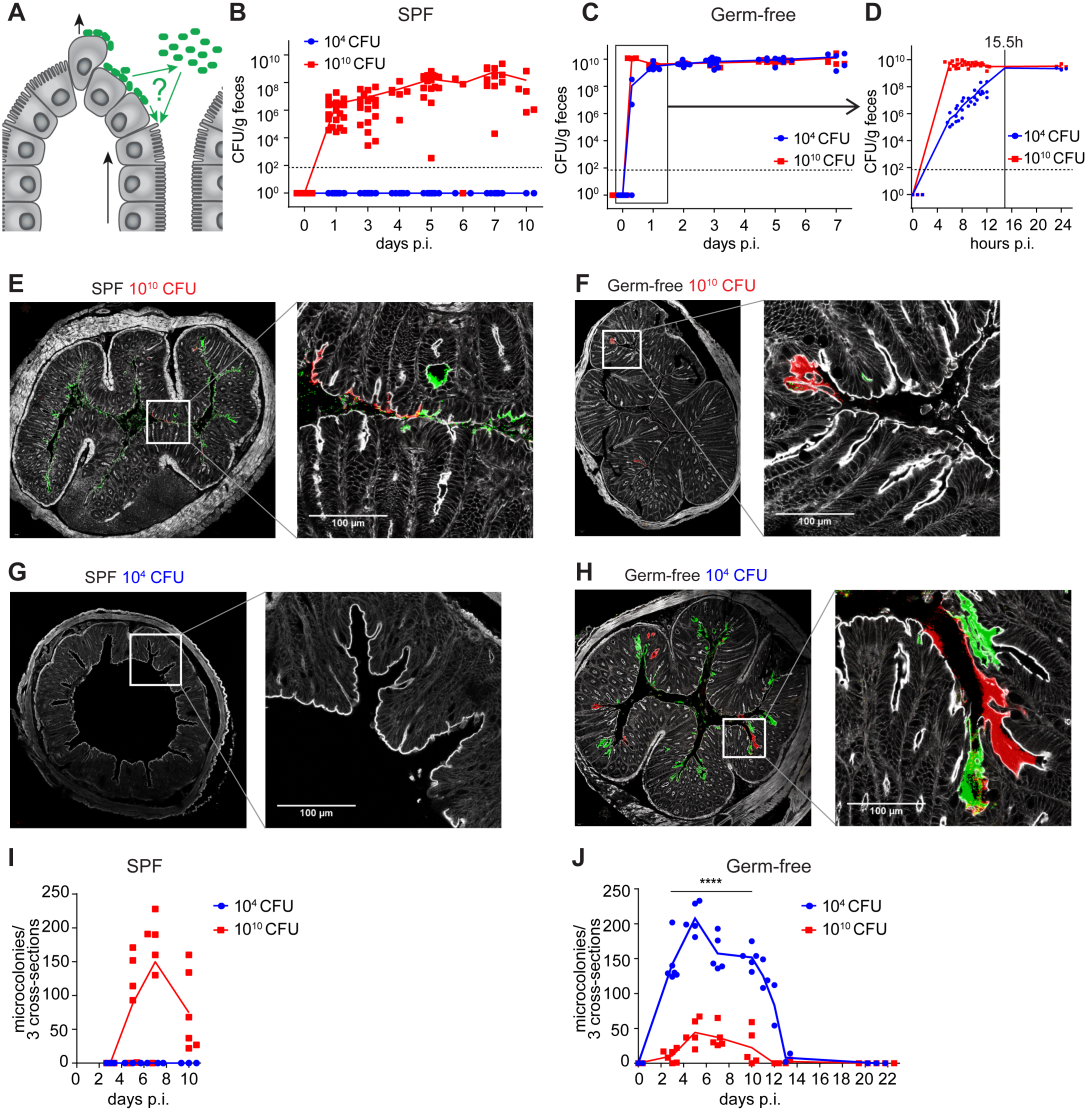
Dynamics of *Citrobacter rodentium* infection in SPF and germ-free wild-type mice. (**A**) Schematic of possible routes of epithelial infection shaping A/E lesion development and renewal in the face of continuous epithelial regeneration. Black arrows indicate direction of epithelial cell migration and luminal exfoliation. Green arrows indicate possible routes of bacterial infection, with or without luminal planktonic stage. (**B-D**) Luminal colonization quantitated by bacterial plating from feces of SPF mice (panel B; n = 12–21 per group and time point) and germ-free mice (panel C; n = 3–15 per group and time point) following inoculation with 10^10^ (red squares) and 10^4^ (blue circles) CFU/mouse, respectively. (D) Early colonization in mice (n = 4 per group) sampled every hour during the first 12 hours after gavage. Fitted exponential curves were used to extrapolate the time to reach levels of 2.5x10^9^ CFU/g in the animals inoculated with 10^4^ CFU. The average of these 4 values is represented by the vertical dotted line (at 15.5 h). (**E-H**) Representative fluorescent microscopy images of distal colon cross sections of SPF (E and G) and germ-free (F and H) mice infected with 10^10^ (E and F) and 10^4^ (G and H) CFU/mouse of *C*. *rodentium* analyzed on day 7 post infection. All individual mice depicted were infected with a 1:1 mixture of bacteria carrying a mCherry (red) or GFP (green) fluorescent protein expression plasmid. Grey, F-actin stained with phalloidin; green, GFP-expressing *C*. *rodentium*; red, mCherry-expressing *C*. *rodentium*. Inset indicates area shown in higher magnification panel. Scale bars: 100 μm. (**I**) Numbers of A/E microcolonies in the distal colon of SPF mice infected with either 10^4^ (blue circles) or 10^10^ (red squares) CFU of *C*. *rodentium* quantified over a time course of 10 days (n = 3–6 per group and time point, data pooled from 2 independent experiments). Connecting lines indicate means. (**J**) Numbers of A/E microcolonies in the distal colon of germ-free mice infected with either 10^4^ (blue circles) or 10^10^ (red squares) CFU of *C*. *rodentium* quantified over a time course of 22 days (n = 2–6 per group and time point, data pooled from 3 independent experiments). Connecting lines connect means; horizontal dotted lines indicate detection limit; ****, p < 0.0001 (Student`s t-test); F-test, Fisher’s test.

Disease severity and mucosal pathology elicited by *C*. *rodentium* correlate with the extent of A/E microcolony formation. The intestinal epithelium protects its structural integrity through rapid cellular renewal driven by crypt stem cell proliferation and crypt-to-villous migration of differentiated epithelial cells, with a complete crypt turnover time of typically 3–5 days in the healthy mouse [6]. Epithelial A/E infection with *C*. *rodentium* lasts for 2–3 weeks. Thus, to compensate for the associated continuous exfoliation of infected cells, A/E microcolonies must also be dynamic and able to renew by continuous epithelial re-infection. The route of this re-infection, required for sustained epithelial A/E pathogenesis, and the dynamics of A/E lesion development remain largely unknown. Re-infection by planktonic luminal colonizers or bacteria shed from A/E microcolonies, or local spread of sessile A/E lesion-associated bacteria without planktonic phase may all contribute (Fig 1A).

All A/E pathogens share the intestinal virulence determinant “locus of enterocyte effacement” (LEE), a genomic island that is essential for A/E lesion formation [3]. LEE encodes a type 3 secretion system that translocates a cocktail of type 3 effector proteins into enterocytes, including Tir (Translocated intimin receptor) that inserts into the enterocyte plasma membrane and functions as adhesion receptor for the LEE-encoded outer membrane protein intimin. The Tir-intimin interaction triggers actin rearrangements that cause the epithelial microvilli to vanish (“effacement”) and pedestal-shaped membrane structures to emerge underneath the attached bacteria [3,7]. A/E virulence expression is regulated at the level of LEE transcription through master regulator Ler (encoded in LEE) that is in turn subject to a complex regulatory network that responds to intestinal environmental factors [3,8]. *Ler*-deficient bacteria are consequently avirulent [7].

Resolution of mucosal *C*. *rodentium* A/E pathology within approximately 21 days of infection depends on functional B and CD4^+^ T cells [9,10]. Furthermore, IL-17-producing Th17 cells, IL-22 production by type 3 innate lymphoid cells (ILC3) and Th22 CD4^+^ effector T cells have all been shown to contribute to *C*. *rodentium* infection control [4,5]. A complex gut microbiota acts as a biological barrier against intestinal *C*. *rodentium* colonization [11,12] and is necessary to clear *C*. *rodentium* from the intestinal lumen once mucosal A/E lesions and inflammation have resolved [13]. However, even though germ-free animals cannot clear *C*. *rodentium* from the gut lumen, they can survive and resolve *C*. *rodentium* mucosal pathology with normal kinetics (within approximately 21 days), by virulence-neutralizing adaptive humoral immune responses [13,14].

In wild-type animals, *C*. *rodentium*-induced A/E pathology progresses slowly with marked intestinal pathology not developing until after 4–5 days of infection. This delay has been assumed to result from microbiota-dependent colonization resistance decelerating intestinal colonization. Disease onset and severity are also strongly determined by innate immunity. MyD88 knockout [15,16] and MyD88-Trif double knockout mice in particular [17], which are deficient in Toll-like receptor (TLR) and IL-1 receptor (IL-1R) superfamily signaling, develop exacerbated and accelerated A/E pathology.

Previous studies have predominantly focused on the acute and resolution phases of infection. Little is known about the nature and importance of host-bacteria interactions occurring during the first hours of infection. In the present study we focused on the early dynamics and control of A/E lesion development and show that the initiation of colonic *C*. *rodentium* microcolonies is limited to a very early stage of infection. These early-established, clonally-renewing A/E lesions trigger a local innate immune response that protects the epithelium from *de novo* A/E lesion formation by luminal bacteria and thus critically determine disease severity and outcome.

## Results

### Clonal intestinal A/E lesion development

To track and quantify *C*. *rodentium* microcolony development *in vivo* over the course of infection, we first inoculated SPF wild type mice with GFP (green fluorescent protein)- and mCherry (red fluorescent protein)-expressing *C*. *rodentium* wild-type bacteria. Microscopic analysis of animals that had been inoculated with 10^10^ CFU of a 1:1 mixture of GFP- and mCherry-tagged bacteria at day 7 post infection revealed that only single-colored microcolonies formed in the colon (Fig 1E), showing that they develop by local clonal expansion (that is, they are derived from the local proliferation of single bacteria; Fig 1E). The fact that many of these clonal microcolonies span several neighboring epithelial cells shows that luminally colonizing bacteria do not contribute to the epithelial re-infection required for the maintenance of established epithelial A/E lesions; otherwise an amalgamation between green and red bacteria over time or a random mosaic of green and red fluorescent bacteria-infected epithelial cells would be expected, neither of which could be observed over the entire course of infection. Thus, colonic A/E lesions trace back to single bacteria and appear to depend on local cell-to-cell spread and not continuous re-seeding from planktonic luminal colonizers to compensate for epithelial shedding. These data further reveal that the epithelium-adherent population of *C*. *rodentium* is derived from a very small founder population (one bacterium per microcolony) and therefore subject to a population bottleneck.

### Clonal A/E lesion development is microbiota status-independent

We next addressed whether this population bottleneck is caused by the colonization barrier conferred by the gut microbiota. Murine *C*. *rodentium* infection is subject to intestinal colonization resistance conferred by a complex gut microbiota through metabolic competition for simple sugars [12,13]. In agreement with this previous work, we found that SPF animals were completely protected against colonization with an inoculum of 10^4^ CFU (Fig 1B), whereas mice inoculated with a very large inoculum of 10^10^ CFU became consistently, albeit still variably, colonized with mean fecal densities that gradually increased to reach a plateau by around day 5 post infection (Fig 1B).

We hypothesized that microbiota-mediated metabolic competition not only impacts on intestinal luminal colonization density but also restricts epithelial colonization and A/E pathogenesis. Colonization resistance may be responsible for the observed population bottleneck in the A/E lesion-associated bacteria through restriction of bacterial access to the epithelium or limitation of growth and survival of attached bacteria, either through direct metabolic competition or by maturing the epithelium and inducing protective non-specific immunity. To test this hypothesis, we studied germ-free mice infected with different doses of *C*. *rodentium*. As expected, in germ-free mice fecal bacterial populations rapidly and invariably reached high densities of between 10^9^ and 10^10^ CFU/g, regardless of inoculum dose (Fig 1C and 1D; S1A Fig). However, the striking clonality of the A/E lesions under SPF conditions was precisely phenocopied in germ-free mice, and was hence independent of microbiota-associated luminal colonization resistance or the effects of microbiota on mucosal immunity (Fig 1F and 1H). Taken together, these experiments revealed that the population bottleneck of early *C*. *rodentium* infection is mediated independently of microbiota-mediated effects.

We next quantitated the numbers of colonic epithelial A/E microcolonies in germ-free and SPF animals over time by fluorescence microscopy (Fig 1I and 1J). Microcolony numbers correlated well with the fraction of infected epithelial area measured by computational image analysis [18] (supplementary S1C and S1D Fig). Unlike in colonization resistant SPF mice (Fig 1E, 1G and 1I), the infection of germ-free mice with a wide range of inoculum sizes (ranging from 10^2^ to 10^8^ CFU) led to peak numbers of colonic A/E lesions that were similar to those observed in SPF mice infected with 10^10^ CFU (Fig 1J and S1B Fig). Thus, in absence of colonization resistance, also lower inocula led to efficient epithelial A/E infection.

### Persistently reduced A/E lesion development associated with very high inocula

**Fig 2:**
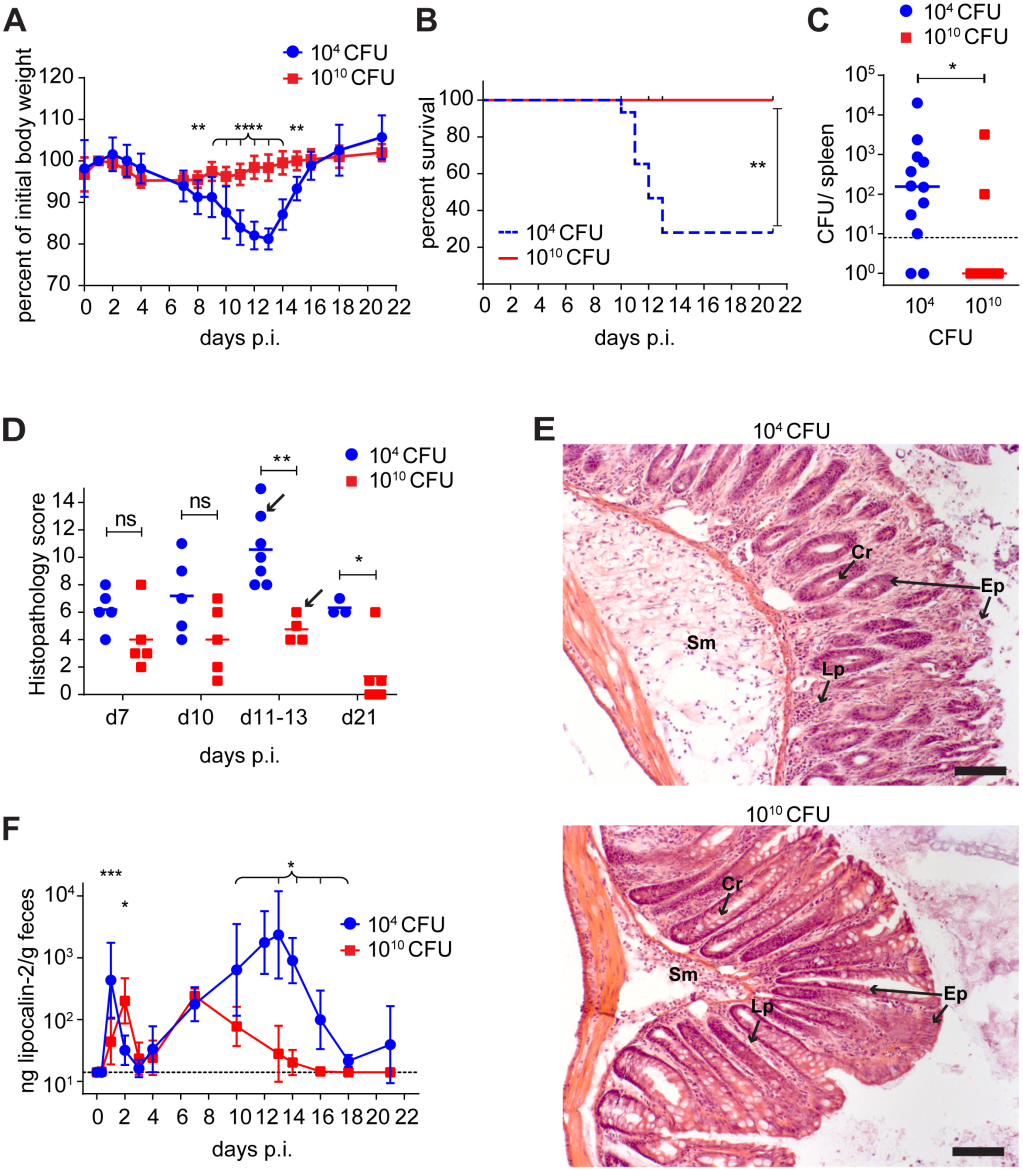
High-dose *C*. *rodentium* infection is associated with reduced severity of disease in germ-free wild-type mice. (**A**) Body weight of germ-free mice infected with either 10^4^ (blue circles) or 10^10^ (red squares) CFU/mouse of *C*. *rodentium*, (n = 15 until day 10). (**B**) Survival curve of the animals shown in A. (**C**) Splenic bacterial loads of mice shown in A infected with 10^4^ (blue circles) or 10^10^ (red squares) CFU/mouse of *C*. *rodentium* for 10–13 days; n = 10–12 per group. (**D**) Histopathological scores of mice shown in panel A (Endpoint criterion: weight loss of >20 %). Mice were scored for epithelial hyperplasia and integrity, infiltration of PMNs, submucosal edema, and loss of goblet cells. Graph shows combined score (= sum of 5 individual scores). (**E**) Representative images of colonic histopathology scored in panel D (H&E staining) of mice infected for 13 days with 10^4^ (top) and 10^10^ (bottom) CFU. Arrows in panel D indicate the individuals depicted. Scale bar: 100 μm; Sm, submucosa; Cr, crypts; Ep, epithelium; Lp, lamina proria; (**F**) Lipocalin-2 measurement by ELISA in feces from infected mice (n = 5 per group, same animals as shown in A; after day 13 n = 3 survivors in 10^4^ group). Error bars indicate standard deviation. Dotted lines indicate detection limit; ns, statistically not significant; *, p < 0.05; **, p < 0.01; ***, p < 0.001; ****, p < 0.0001; statistical tests: Student`s t-test (A), Mantel-Cox test (B), Mann-Whitney U test (C, D, F).

Remarkably however, the inoculation of germ-free mice with the extremely high dose of 10^10^ CFU was associated with significantly *reduced* virulence, leading to significantly lowered numbers of colonic A/E lesions (Fig 1F; compare with Fig 1H). Although the intestinal colonization kinetics in lower-dose versus high-dose infections only differed during the first <16 hours (Fig 1D and S2B Fig), A/E lesion numbers remained significantly decreased over the entire course of infection (Fig 1J). This was associated with significantly improved clinical disease parameters between day 8 and 21, including weight loss (Fig 2A and 2B), systemic bacteremia (manifesting in live recoverability of *C*. *rodentium* from the spleen; Fig 2C), colonic mucosal histopathology (Fig 2D and 2E), and fecal concentrations of lipocalin-2 (a fecal marker for colitis severity; Fig 2F).

We considered two alternative explanations for the mechanism by which the size of the inoculum in germ-free animals could have such a persistent effect on disease severity: First, ongoing bacteria-intrinsic downregulation of virulence in luminal *C*. *rodentium* after reaching high intestinal luminal density, or second, inhibition of ongoing A/E lesion induction by an early *C*. *rodentium*-induced host immune response.

Bacteria-intrinsic virulence regulation is considered important for the *in vivo* fitness of this pathogen [19]. We found that colonic luminal *C*. *rodentium ler* transcription was reduced at 16 hours after inoculation in mice that had been inoculated with 10^10^ CFU compared to mice inoculated with only 10^4^ CFU (S2A and S2B Fig). The main difference between the infection with an inoculum of 10^10^ CFU and that with lower inocula is in the early population dynamics *in vivo*. Upon inoculation, bacterial populations enter an early logarithmic growth phase followed by a steady-state stationary phase (see Fig 1D). In germ-free mice inoculated with 10^4^ CFU, the logarithmic growth phase lasts for approximately 15.5 h (Fig 1D). In germ-free mice inoculated with 10^10^ CFU, the bacteria lack this growth phase (Fig 1D). The associated colonic virulence gene expression differences we measured at 16 hours after inoculation could explain a delayed disease onset, but are an unlikely sole explanation for the maintained reduction in A/E lesion burden, as it is known that fully colonized germ-free animals remain luminally colonized with phenotypically virulent (*ler*-expressing) bacteria for at least 7 days [13]. Moreover, population density-dependent virulence regulation by the *croIR*-encoded acyl homoserine lactone (AHL) type quorum sensing system, reported previously to regulate bacterial adhesion and virulence negatively (in an *ler*/LEE-independent manner) at high population density [20], did not explain our observations. Infection with 10^10^ CFU of a quorum sensing-deficient *croI* mutant reproduced the reduced A/E lesion density phenotype (S2D Fig).

**Fig 3:**
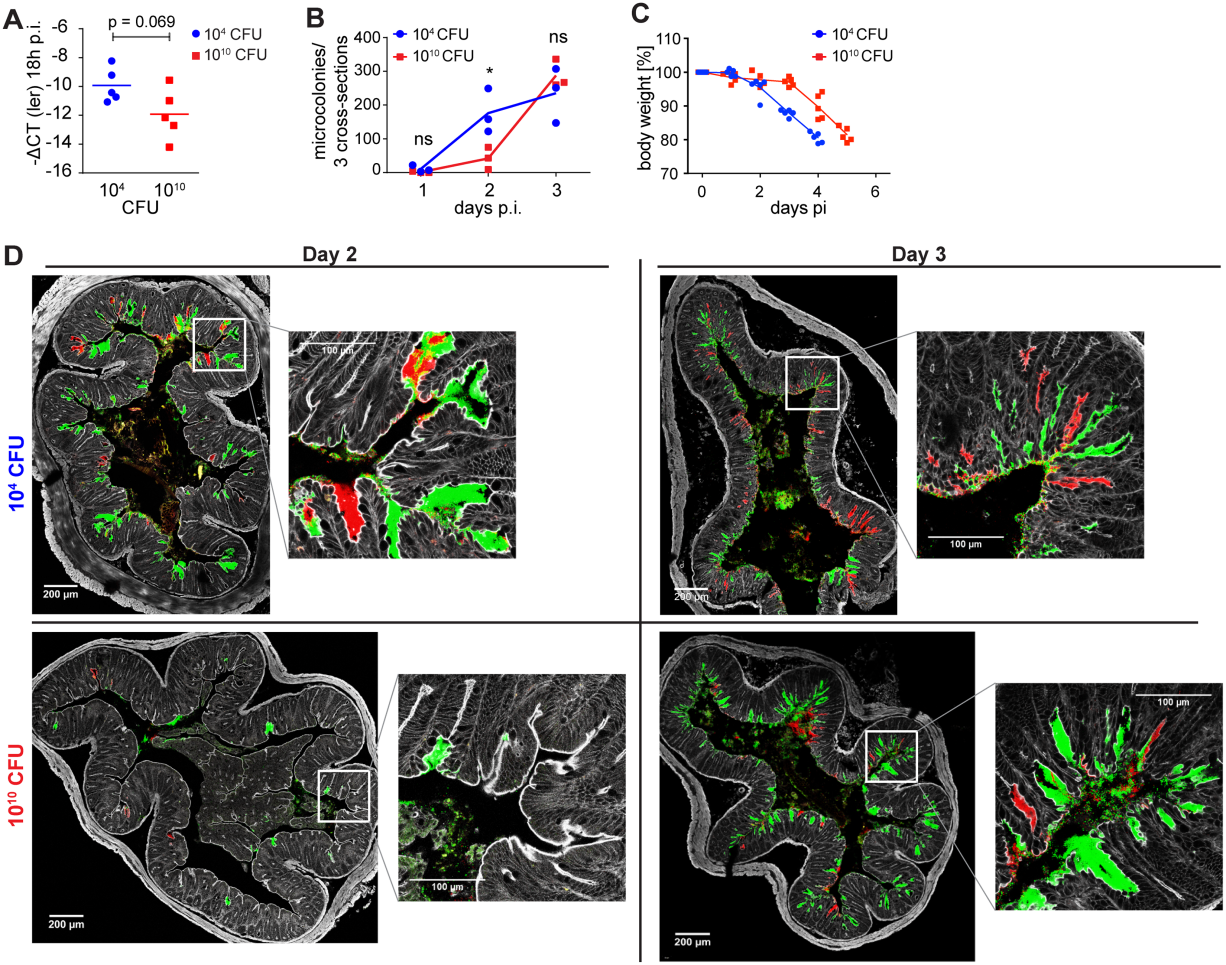
High-dose *C*. *rodentium*-infected MyD88/Trif-deficient mice do not display persistent disease attenuation. (A-C) Germ-free MyD88^-/-^Trif^lps/lps^ mice were infected with either a low (10^4^ CFU, blue dots) or a high dose (10^10^ CFU, red squares) of wild type *C*. *rodentium*. (**A**) qPCR quantification of bacterial *ler* mRNA in colon content of infected MyD88^-/-^Trif^lps/lps^ germ-free mice at time point 18 h. Expression of *ler* was normalized to the mRNA encoding bacterial housekeeping gene *rpoD*. (**B**) Microcolonies were quantitated in the distal colon after 1, 2, or 3 days of infection. (n = 3 per group, pooled from two independent experiments). (**C**) Body weights of germ-free MyD88^-/-^Trif^lps/lps^ mice infected with either a low (10^4^ CFU, blue circles) or a high dose (10^10^ CFU, red squares) of wild type *C*. *rodentium* were monitored daily until reaching ethical endpoint. Symbols represent individual mice. (**D**) Representative fluorescent microscopy images of distal colon of germ-free MyD88^-/-^Trif^lps/lps^ mice after infection with doses of 10^4^ or 10^10^ CFU of *C*. *rodentium* for 2 days (left) and 3 days (right). Every individual depicted was infected with a 1:1 mixture of bacteria carrying a mCherry and GFP expression plasmid, respectively. Grey, F-actin/phalloidin; green, GFP-expressing *C*. *rodentium*; red, mCherry-expressing *C*. *rodentium*. Scale bars: 200 μm and 100 μm as indicated. ns, statistically not significant; *, p < 0.05; Student`s t-test.

In support of a host-mediated mechanism, however, we found that grossly innate immunocompromised germ-free Myd88/Trif double-deficient mice [21], unlike wild-type animals (Figs 1F, 1H, 1J and 2), did not show a persistent disease attenuation following infection with 10^10^ CFU of *C*. *rodentium*. Even though the reduction of *ler* expression following infection with 10^10^ CFU reproduced in MyD88/Trif-deficient mice (Fig 3A) and was associated with a transiently reduced extent of colonic A/E microcolony formation on day 2 post infection, microcolony numbers were no longer different on day 3 post infection (Fig 3B and 3D). In an independent experiment, the weight loss of infected MyD88/Trif double-deficient germ-free mice was followed until animals reached a defined ethical endpoint (≤ 80% initial body weight). Mice that were infected with 10^10^ CFU continued to lose weight at a rate similar to mice infected with 10^4^ CFU, but with a delay of approximately 24 hours (Fig 3C). These data suggest that an early MyD88/Trif-dependent immune response restricts the ongoing induction of A/E lesions, rather than the maintained downregulation of bacterial virulence.

MyD88-/Trif double-knockout mice are deficient for TLR as well as IL-1R family receptor signaling. To investigate the role of TLR- and inflammasome-related pro-inflammatory signaling, we analyzed germ-free Caspase-1/11-deficient mice. Although these mice have been described as hyper-susceptible to *C*. *rodentium* infection [22], we found that they developed a persistently attenuated A/E pathogenesis in response to an infection with 10^10^ CFU of *C*. *rodentium*, similar to wild-type mice (S3 Fig). Thus, mucosal innate immune activation through MyD88/Trif-TLR-signaling determines severity of ongoing A/E pathogenesis in an early phase of infection.

Taken together, our data indicated that an early host innate immune response is induced at an early stage of infection and determines ongoing disease severity by limiting the induction of A/E lesions to an early window of opportunity. The remaining course of infection would then be driven by the early induced and persisting A/E lesions. A testable prediction of this hypothesis is that *C*. *rodentium-*infected wild-type animals should become insensitive to superinfection with a congenic strain of *C*. *rodentium* after the postulated window of opportunity.

### Induced innate immunity limits A/E lesion induction to an early window of opportunity

**Fig 4:**
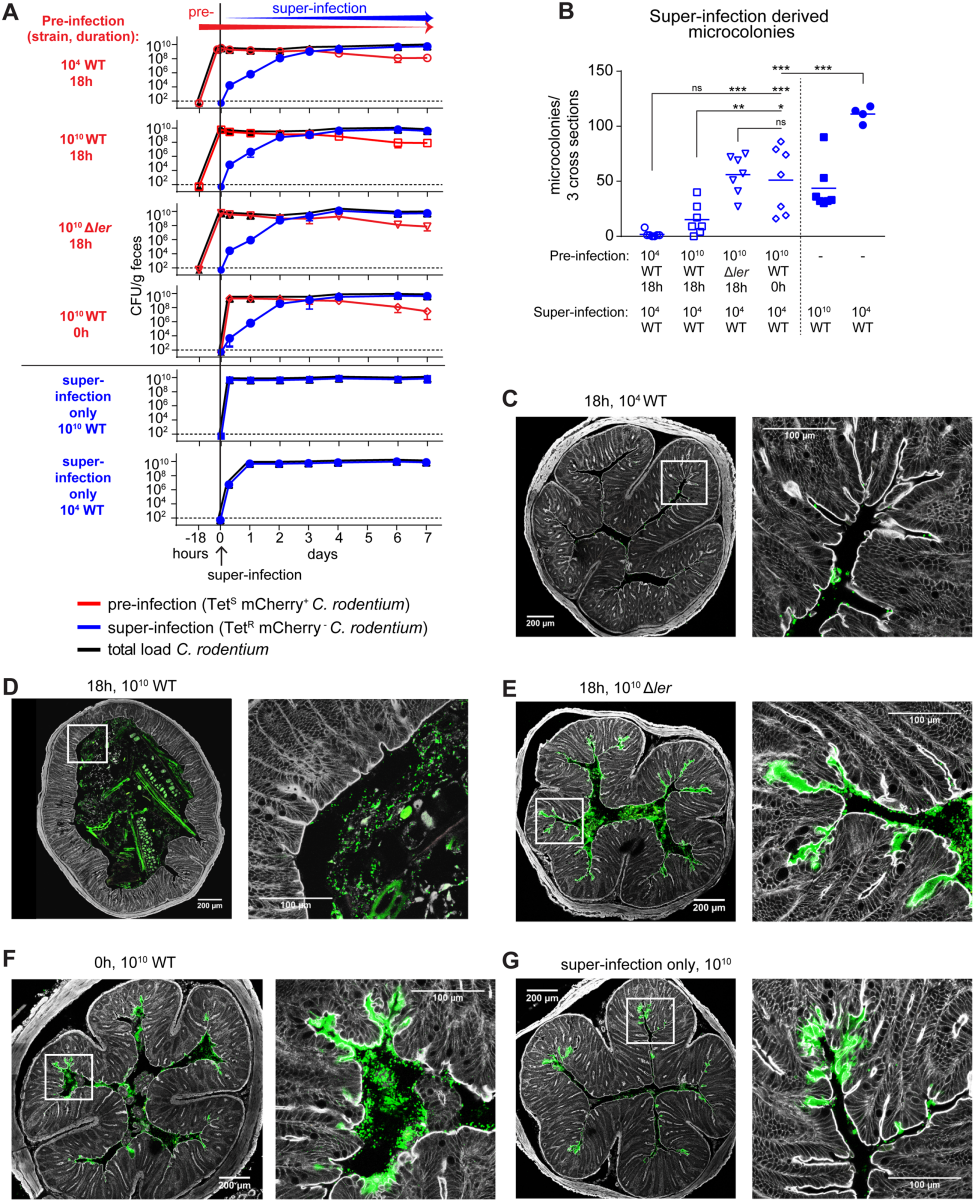
An early window of opportunity for A/E microcolony induction in wild-type mice. (**A**) Colonic luminal colonization trajectories of the pre-infection strain (red open symbols: circles, 10^4^ CFU Wild type *C*. *rodentium* [WT]; squares, 10^10^ CFU WT; inverted triangles; 10^10^ CFU Δ*ler* mutant), the super-infection strain (blue filled symbols: circles, 10^4^ CFU WT; squares, 10^10^ CFU WT), and total bacteria (pre-infection + super-infection strain; black triangles). Connecting lines connect means; error bars indicate standard deviations. Horizontal dotted lines indicate the lower detection limit. (**B**) Microcolony formation of superinfection strain after 7 days. N = 6–7 per group, pooled from 3 independent experiments. (**C-G**) Representative confocal fluorescent microscopy images of distal colon of mice shown in panel B. Grey, F-actin/phalloidin; green, anti-O-antigen Antibody (*C*. *rodentium* total); red, mCherry-expressing *C*. *rodentium* (pre-infection strain only); Scale bars: 200 μm and 100 μm as indicated; White squares indicate origin of higher-magnification image; *, p < 0.05; **, p < 0.01; ***, P < 0.001; ns, not significant (p ≥ 0.05); statistical test: one-way ANOVA.

To test this hypothesis, we developed a strain combination infection-superinfection model, in which two differentially tagged *C*. *rodentium* strains could be administered sequentially. This allowed us to elucidate the effect of the infection with the first strain on the pathogenesis of the second and how this depended on bacterial genotype, host genotype, and time interval between primary and superinfection (see schematic in Fig 4A). The aim of this approach was (i) to demonstrate and map the duration of the proposed infection window of opportunity directly, (ii) to show definitively the inhibition of A/E lesion initiation by luminal bacteria outside the window of opportunity, and (iii) to differentiate between bacterial and host contributions to its induction.

Germ-free mice were pre-infected with a kanamycin-resistant (Kan^R^) mCherry-tagged *C*. *rodentium* strain (wild-type or *ler* mutant), followed 18 hours later by super-infection (or in the negative-control group co-infected) with 10^4^ CFU of an isogenic tetracycline-resistant (Tet^R^) wild-type strain (schematized and data shown in Fig 4A). All groups were treated orally with the bacteriostatic antibiotic tetracycline starting 3 h prior to the superinfection to shift colonization from the Tet-sensitive primary strain to the Tet-resistant super-infecting strain. The luminal colonization kinetics of each strain were determined by selective bacterial plating from feces (Fig 4A). On day 7 after superinfection the animals were sacrificed and colonic A/E lesions (all of which were derived from super-infection, and therefore mCherry-negative) were quantified after labeling of *C*. *rodentium* with an anti-*C*. *rodentium* LPS antibody (Fig 4B–4G).

Super-infection was influenced by bacterial-bacterial competition between pre-and super-infecting bacteria, since there was overlap between pre- and super-infection. The co-infection control in which pre- and super-infection inocula were administered simultaneously (Fig 4, “10^10^ WT 0h”) consequently presented with 2.2±3.7 -fold (mean±SD) reduced numbers of colonic A/E lesions compared to the 10^4^-CFU-super-infection-only control (Fig 4B). This effect reduced A/E lesion density to values similar to those observed in the super-infection-only control inoculated with 10^10^ *C*. *rodentium* (Fig 4, “super-infection only 10^10^ WT). However, 18-hour pre-infection with 10^10^ wild-type *C*. *rodentium* significantly reduced the A/E lesion formation a further 3.3±0.3 -fold relative to the co-infection control (Fig 4B and 4D). Remarkably, 18-hour pre-infection with an isogenic avirulent *ler* mutant had no such additional protective effect (Fig 4B and 4E), showing that LEE-dependent (*ler* regulated) pathogenesis in the first 18 hours of pre-infection induced epithelial resistance to A/E lesion induction. Moreover, the effect was even more pronounced when pre-infecting with 10^4^ (near-full protection, more than 25-fold reduced A/E lesion relative to co-infection control; Fig 4B and 4C) instead of 10^10^ wild-type *C*. *rodentium*, indicating that the epithelial resistance induction by pre-infection correlated with infectiousness rather than bacterial load of the pre-infection inoculum.

**Fig 5:**
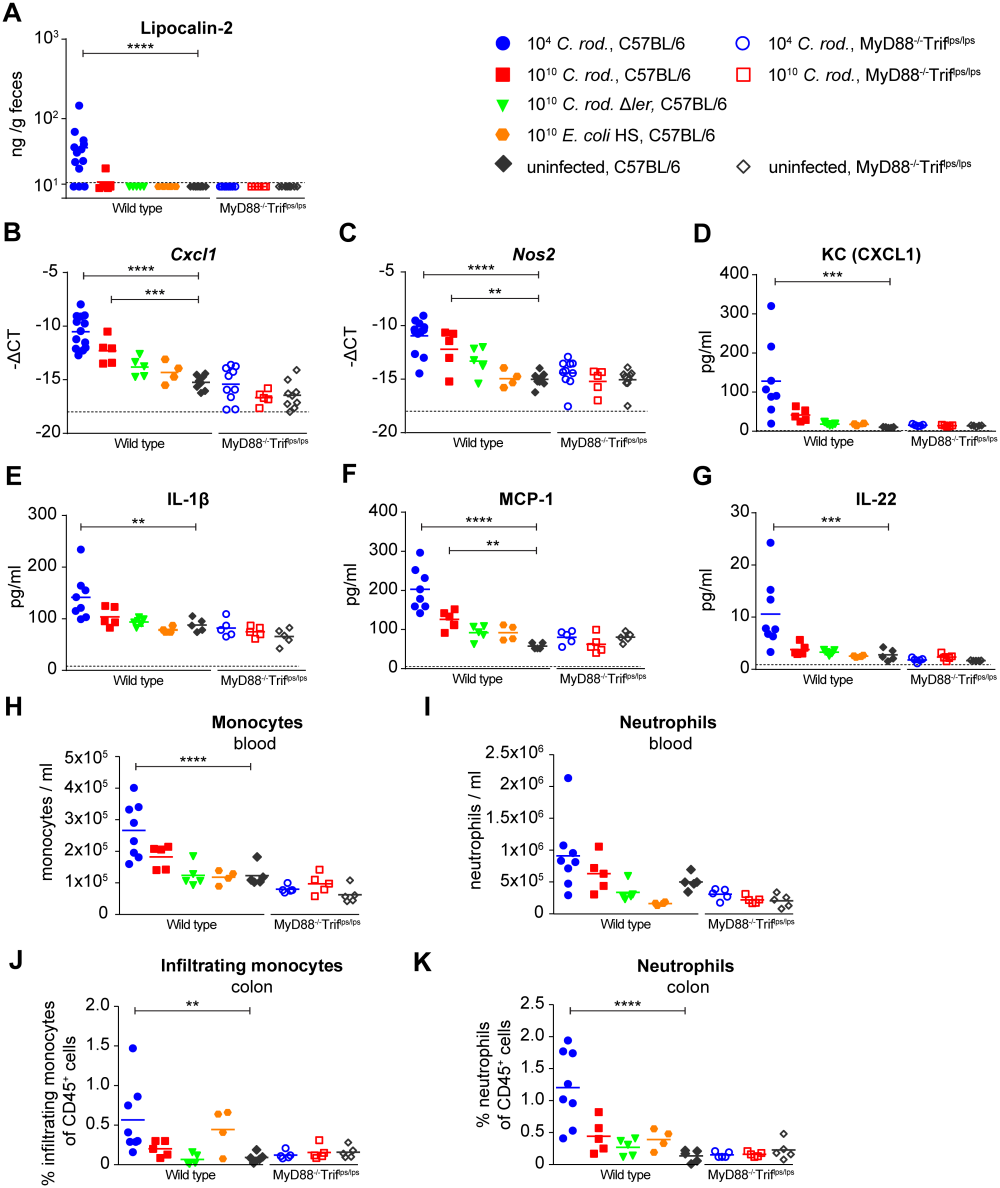
Early *C*. *rodentium*-induced innate immune response. (**A**) Lipocalin-2 quantification by ELISA in feces of germ-free mice infected for 18 h with 10^4^ CFU *C*. *rodentium* (blue circles), 10^10^ CFU *C*. *rodentium* (red squares), 10^10^ CFU *C*. *rodentium* Δ*ler* (green triangles), 10^10^ *E*. *coli* HS (orange hexagons) or left uninfected (grey diamonds). MyD88^-/-^Trif^lps/lps^ germ-free mice were infected for 18 h with 10^4^ CFU *C*. *rodentium* (open blue circles), 10^10^ CFU *C*. *rodentium* (open red squares), or left uninfected (open grey diamonds). N = 4–13 per group. Data were pooled from 3 independent experiments. (**B and C**) Gene expression levels of *Cxcl1* (B) and *Nos2* (C) in the distal colon of infected mice as determined by qPCR. (**D-G**) Cytokine protein levels in the distal colon of infected mice as determined by Luminex technology. (**H and I**) Leukocyte analysis by flow cytometry of peripheral blood. Monocytes were defined as CD45^+^, CD11b^+^, CD115^+^ population (H). Neutrophils were defined as CD45^+^, CD11b^+^, Ly6G^+^ population. Quantities are expressed as absolute numbers of cells per mL blood. (**J and K**) Large intestinal leukocyte analysis by flow cytometry. Infiltrating monocytes were defined as CD45^+^, CD11b^+^, CD64^-^, LygG^-^, Ly6C^high^, and MHCII^-^ population (J). Neutrophils were defined as CD45^+^, CD11b^+^, Ly6G^+^ population (K). Quantities are expressed as percentages of leukocytes (of CD45^+^ population). Dotted lines represent detection limit. ns, statistically not significant; *, p < 0.05; **, p < 0.01; ***, p < 0.001; ****, p < 0.0001; groups were compared with the matching uninfected control group; statistical tests: Kruskal-Wallis (A), and 1-way ANOVA (B-K).

**Fig 6:**
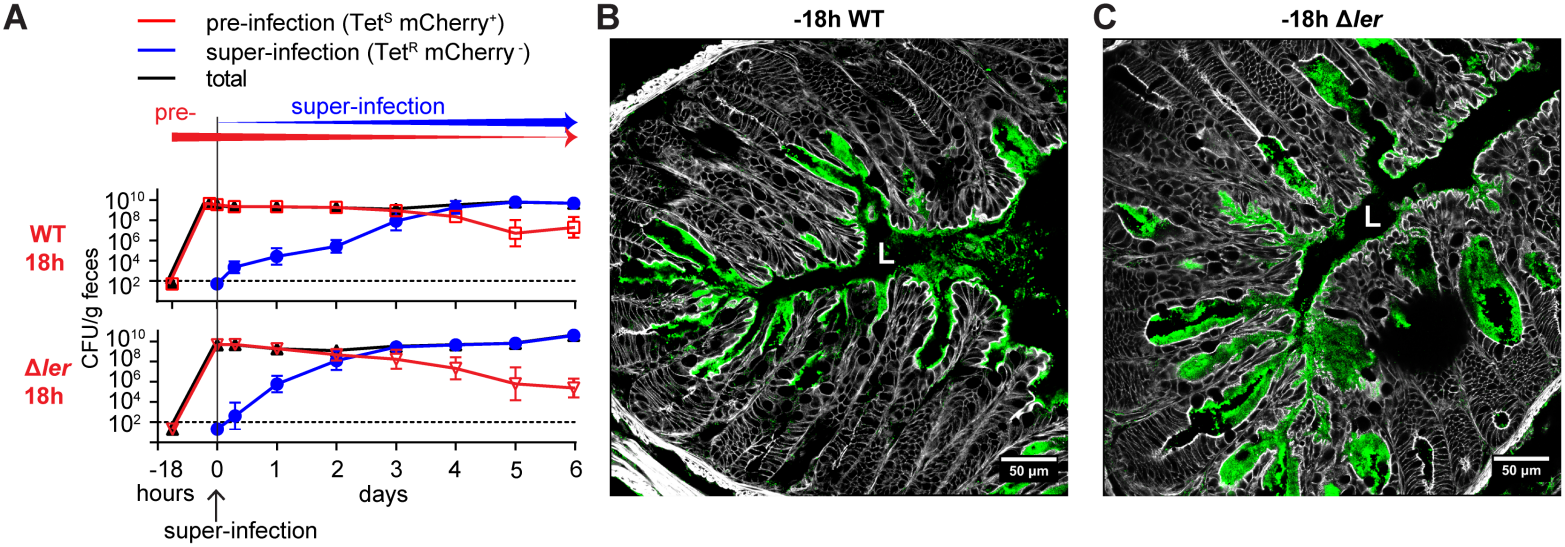
Ongoing A/E lesion induction in mice deficient for innate signaling through MyD88 and Trif. (**A**) Colonic colonization dynamics in MyD88^-/-^Trif^lps/lps^ germ-free mice consecutively infected, first with mCherry^+^ Kan^R^ Tet^S^ *C*. *rodentium* wild type (WT) or an isogenic Δ*ler* mutant (10^10^ CFU, red squares and triangles), and 18 hours later superinfected with mCherry^-^ Kan^S^ Tet^R^ *C*. *rodentium* wild type (10^4^ CFU, blue circles). Total *C*. *rodentium* counts are depicted as black symbols. Connecting lines connect means; error bars indicate standard deviation; horizontal dotted lines indicate detection limit. (**B and C**) Representative fluorescent microscopy images of distal colon of MyD88^-/-^Trif^lps/lps^ mice shown in panel A pre-infected with wild type (B) and Δ*ler* (C) *C*. *rodentium*, respectively. Grey, F-actin/phalloidin; green, anti-*Citrobacter* O-antigen antibody (pre-infection strain and superinfecting strain); red, mCherry-expressing *C*. *rodentium* (pre-infection strain only; none detectable). Scale bars: 50 μm.

This early mucosal resistance phenotype was associated with an early increase of mucosal protein levels of IL-1β, IL-22, KC/CXCL1 and MCP-1 (Fig 5D–5G) and elevated transcription rates of *Cxcl1* and *Nos2/iNos* (Fig 5B and 5C) measured at 18 hours post infection. At the same time point we observed the increased recruitment of neutrophils and monocytes into blood and lamina propria, associated with an early increase of lipocalin-2 (mainly produced by neutrophils) detectable in the feces (Fig 5A and 5H–5K). Colonic histological analysis confirmed that these changes occurred prior to the onset of acute colonic histopathology (S4 Fig), although the response markers measured may overlap with those associated with the acute A/E lesion-associated inflammation developing later. These responses were induced specifically by virulent *C*. *rodentium*, but not an avirulent *ler* mutant or the commensal *E*. *coli* strain HS. Thus, the virulent *C*. *rodentium*-induced resistance to epithelial A/E superinfection with luminal bacteria was associated with an early mucosal innate immune response. It was more potently induced by a low-dose (10^4^ CFU) than a high-dose (10^10^ CFU) infection with wild-type *C*. *rodentium*. Hence, LEE/ler-dependent *C*. *rodentium* virulence, rather than unspecific bacterial innate immune receptor agonists like LPS (expected to be increased upon administration of 10^10^ bacteria and similar between *C*. *rodentium* and *E*. *coli*), appears to be the main driver of this response. Finally, the response was abrogated in MyD88-/Trif-double-deficient mice (Fig 5, **open symbols**). MyD88/Trif double-deficient mice consequently lacked protection against epithelial superinfection, with no protective effect of wild-type *C*. *rodentium* pre-infection over the control pre-treatment with a Δ*ler* mutant (Fig 6A–6C). The specific cellular and molecular innate immune components necessary and sufficient for this protective mechanism remain to be identified.

Overall, our data show that mucosal innate immunity, specifically activated by virulent *C*. *rodentium* in an *ler*/LEE-driven and TLR-signaling dependent manner, very effectively limits the establishment of A/E pathogenesis of *C*. *rodentium* to a window of opportunity of <18 hours duration that determines ongoing geography and severity of infection.

## Discussion

We show in this paper that the development of colonic *C*. *rodentium* A/E pathogenesis is critically dependent on very early bacteria-host interactions. The characteristic A/E lesions that determine the severity of infection originate from a limiting number of single-bacterial epithelial infection events that need to occur within the first 18 hours of infection. The ongoing infection is driven by the early induced and locally growing epithelial A/E lesions that counteract epithelial shedding by continuous localized epithelial reinfection. Whilst this localized epithelial re-infection allows A/E lesions to persist on the epithelium for more than 10 days, until an adaptive immune response clears the infection, the window of opportunity for their induction by planktonic bacteria present in the lumen is limited to the first 18 hours. This is remarkable because the colonic lumen is known to remain colonized by phenotypically virulent bacteria for at least a week [13] and these bacteria are assumed to be more infectious than *in-*vitro-grown bacteria, which is important for effective fecal-to-oral transmission [23]. Restriction of A/E lesion induction to such an early time window is due to an early host response specifically induced by virulent *C*. *rodentium*, but not by an avirulent *C*. *rodentium* mutant. Similarly, mucosal stimulation with the related Gram-negative commensal *E*. *coli* could not phenocopy the observed phenomenon. Also the physiologic mucosal conditioning with a complex microbiota does not protect against epithelial A/E pathogenesis, but only impedes intestinal colonization with this pathogen. The abolition of the pathogen-induced mucosal resistance by a deficiency in MyD88-/Trif-signaling and superinfection experiments clearly show that an innate host response, rather than bacteria-intrinsic virulence gene regulation or bacteria-bacteria interaction, is mechanistically responsible. The specific cellular and molecular innate immune components required and sufficient for this protective effect remain to be identified. Whilst highly effective against induction of A/E lesions, the MyD88-/Trif-dependent innate immunity is not sufficient to abolish the localized re-infection occurring around persisting A/E lesions as well, although it may limit the rate of local spread of A/E lesions across the epithelial surface.

Notwithstanding the fact that early host immunity is the main driver of the phenomenon described, the bacteria-intrinsic regulation of *C*. *rodentium* virulence gene expression is known to be central to the pathogenesis of this species. The altered virulence of *C*. *rodentium* following an inoculation in unphysiologically high numbers of 10^10^/dose into germ-free animals provided us with an experimental tool to demonstrate the importance of bacterial virulence during the critical early phase of infection. In this context individual A/E lesions developed normally but in persistently reduced number, with extended areas of surface epithelium remaining uninfected. The ongoing epithelial re-infection that must occur to compensate for the effect of epithelial shedding thus appears to have a remarkably short range and may depend on epithelial adherence that is already established. Exact elucidation of this specialized re-infection mechanism is beyond the scope of the present study but warrants future efforts.

Our work in gnotobiotic models demonstrates that colonic A/E lesions induced by *C*. *rodentium* in an early phase of the infection—in immunocompetent mice—limit the ongoing induction of new A/E lesions. As a consequence the survival and localized renewal of early-induced A/E lesions, rather than the continued exposure to virulent bacteria in the environment or intestinal lumen is the main determinant of infection severity. This was unexpected, as the virulent bacteria shed into the colonic content and consequently the feces have been shown previously to have a “hyper-infectious” phenotype [23]. However, the situation in conventional, colonization resistant, mice may be more complex: It has been shown that, in SPF mice, *in-vitro*-grown *C*. *rodentium* initially infects and accumulates mainly at the cecal patch and only approximately two days later spreads to the colon, strongly suggesting that longitudinal cecal-to-colonic re-infection can occur [23]. This has been ascribed to an “hyper-infectiousness” phenotype of *C*. *rodentium* acquired during intestinal passage that is associated with the increased expression of LEE and related virulence determinants [24]. It has further been shown by others that virulence is required to efficiently overcome intestinal colonization resistance in SPF mice [7,13]. In contrast to the delayed colonic infection observed with *in-vitro*-grown *C*. *rodentium*, the inoculation of naïve SPF animals with feces from infected mice led to the more direct infection of the colon [23]. It is readily conceivable that in mice receiving an *in-vitro*-grown inoculum, such a “hyper-infectious” bacterial population can build up not only during the initial cecal patch infection observed in SPF mice, but also more rapidly in the non-colonization-resistant germ-free intestine. Whilst our experiments in SPF mice (also using *in-vitro*-grown bacteria) recapitulated the previously described 2-day delay in colonic infection (see Fig 1), the colonic infection kinetics in the germ-free mice was equivalent to that described for SPF mice infected via the fecal-to-oral route. Thus, in absence of microbiota-mediated colonization resistance, cecal patch colonization may not be a prerequisite for colonic infection, but we cannot rule out that cecal infection plays an important role in all models, also independent of colonization resistance.

Our observation that very high inocula of (stationary phase) *C*. *rodentium* in germ-free mice are hypovirulent is relevant for the correct interpretation of earlier work in germ-free animals. For instance, the surprising finding that germ-free mice survive and can clear *C*. *rodentium* infection with similar mucosal immune activation kinetics as SPF mice, described recently [13], was reproduced in our experiments but depended on the infectious dose. We found a very large dose of 10^10^ CFU (most labs use between 10^7^ and 10^9^ CFU) was less virulent in germ-free than in SPF mice. Conversely, we show that an “optimal” dose of 10^4^ CFU is highly pathogenic in germ-free mice, yet below the minimum infectious dose in SPF mice due to microbiota-related colonization resistance. Thus, it is not straightforward to compare *C*. *rodentium* disease kinetics between animals with different microbiota statuses.

Our findings may also be relevant for other intestinal pathogens. Clonal intraepithelial microcolony formation has recently been described for the invasive epithelial pathogenesis of *Salmonella typhimurium* [25,26]. Sellin *et al*. described an early epithelial-intrinsic protective mechanism dependent on activation of epithelial Nod-like receptor NLRC4 and Caspase-1 that leads to elimination of infected enterocytes and curbs epithelial infection within the first hours of infection [25]. *C*. *rodentium* A/E infection has been shown to be more severe in NLRC4- [27] and Caspase-1-deficient mice [22]. Although we found that Caspase-1/11-deficient animals did not phenocopy MyD88-/Trif-deficiency in our analyses, the very early mucosal innate immune response appears to be similarly important in the control of invasive intestinal pathogens.

Further studies are needed to analyze how the new information obtained with the murine *C*. *rodentium* model translates into human A/E infection. EPEC-induced acute diarrhea is primarily a neonatal and infantile infection, whereas adults are relatively protected from natural infection by intestinal colonization resistance and develop disease only after intake of very large inocula (10^8^−10^10^ CFU; [28]). A recent study using a novel neonatal murine EPEC infection model revealed strikingly similar A/E microcolony formation in the neonatal small intestine, and revealed an age-dependent but microbiota-independent resistance of the adult epithelium against A/E lesion formation [29].

Our findings may have several medical implications. First, since the function of LEE is highly conserved between murine and human A/E pathogens, the observed LEE-mediated induction of epithelial resistance to super-infection may also be relevant to human infection. We have identified the initial 18 hours of murine A/E infection as a critical time window for controlling and surviving, or potentially preventing the infection. Even incompletely or transiently neutralizing antibodies may thus be able to substantially reduce intestinal morbidity. As human A/E infection is most severe in newborns and infants, the potential benefit of maternal immunity, protective lactational antibodies and mucosal vaccination will be an important aspect of future work. Remarkably, to the authors’ knowledge it is still unclear to what extent human EPEC infection generates long lasting immunity or neonate-protective maternal antibodies [30], and there is no EPEC vaccine currently available. However, we also need to consider the possibility that specific intestinal antibodies, through dampening the observed rapid innate immune activation, may alter disease progression and even worsen disease outcome.

Second, antibiotic therapy is not a recommended therapy for human A/E infection, and contraindicated in EHEC infection where antibiotic treatment is associated with an increased risk of hemolytic-uremic syndrome. Due to worldwide emergence of multi-resistant *E*. *coli* strains and the negative effect on microbiota-conferred colonization resistance, empiric antibiotic therapy is limited to severe cases. However, our findings in the murine model demonstrate that A/E microcolony formation is sensitive to antibiotic treatment, whilst epithelial re-infection by the incompletely eradicated luminal bacteria or superinfection by resistant bacteria is efficiently inhibited by mucosal innate immunity. Hence, optimized antibiotic regimens or novel selective antimicrobials, such as type 3 secretion system inhibitors [31], may have therapeutic potential in the treatment of EPEC infection.

## Material and methods

### Ethics statement

All animal experiments were performed according to protocols approved by the Bernese cantonal ethical committee for animal experiments and carried out in accordance with Swiss Federal law for animal experimentation (license numbers BE94/11 and BE91/14).

### Animals

All mice had a C57BL/6 background. Germ-free animals were derived and maintained germ-free in flexible film isolators in the Genaxen Foundation Clean Mouse Facility (CMF) of the University of Bern as described [32]. Myd88^-/-^ Ticam1/Trif^*lps/lps*^ mice were kindly provided by Prof B. A. Beutler, The Scripps Research Institute (la Jolla, CA, USA) [33] and derived germ-free as described [21]. Germ-free caspase-1/11-deficient mice [34] were generated by axenic embryo transfer of cryopreserved 2-cell-stage embryos (The Jackson Laboratory, Bar Harbor ME, USA). Experimental germ-free mice were aseptically transferred to autoclaved sealsafe-plus individually ventilated cages (IVCs) under positive pressure (Tecniplast, Italy) in a gnotobiotic barrier unit. Cage changes were carried out under strictly aseptic conditions. In all experiments animals were provided with sterile mouse chow (Kliba 3437; autoclaved) and autoclaved water ad libitum. SPF mice were acquired from Envigo (former Harlan, the Netherlands) and housed in IVCs in the Zentrale Tierställe of the University of Bern.

### *In vivo* infections

To generate contamination-free bacterial inocula, LB medium supplemented with appropriate antibiotics (see section bacterial culture for details) in sterile-filter-sealed flasks was aseptically inoculated from single colonies of the test bacterium and incubated, with shaking at 150 rpm at 37°C for 16 h. Bacteria were harvested by centrifugation (7 min, 4700 × g, 4°C) in a sterile aerosol-proof assembly, washed in autoclaved sterile PBS and concentrated to a density of 5 × 10^10^ CFU/mL in sterile PBS, performed aseptically under a sterile laminar airflow. To generate lower densities cultures were diluted appropriately in PBS. The bacterial suspensions were aseptically aliquoted in autoclaved plastic tubes and sealed in a sterilized secondary containment. The sterile tubes containing the inocula and germ-free mice were aseptically imported into a sterilized laminar flow hood, and each animal inoculated with 200 μL of bacterial suspension by gavage, carried out wearing sterile surgical gowns and sterile surgical gloves. Fresh fecal pellets were collected aseptically, suspended in sterile PBS, and plated in serial dilutions on LB plates containing the appropriate antibiotics and incubated at 37°C for 24 h. Weight of mice was measured using a hand held balance and calculated as a percentage of the body weight in the beginning of the experiment. For tetracycline treatment, mice were gavaged with 200 μL of a sterile tetracycline solution (50 mg/L) containing 2% sucrose, and the same solution was used as drinking water. The tetracycline-water was protected from light and replaced every 3 days.

### Bacterial culture

LB medium (Sigma-Aldrich, Germany) was used as the standard growth media. Where required, the following supplements were added to the media: chloramphenicol (Sigma-Aldrich, China, 6 μg/mL), tetracycline (Sigma-Aldrich, USA, 12.5 μg/mL), kanamycin (Calbiochem, USA, 50 μg/mL). Tetracycline counterselective plates were prepared as described [35,36].

### Bacterial genetic engineering

All bacterial strains and plasmids used or generated in this study are specified in S1 Table. All *Citrobacter rodentium* strains used in this study are derivatives of the wild type strain ATCC51459 (ATCC, Manassas VA). All deletions were carried out by Lambda Red recombineering. Mutagenesis primer sequences are specified in S2 Table. (i) Strain HA526 (Δ*ler*::*tetRA*) was generated by deletion of *ler* using recombineering plasmid pSIM5 as described [36–38]. A *tetRA* recombineering amplicon was amplified from genomic *tetRA* template DNA (isolated from a Tn10-containing bacterial strain) with primers CR*-ler*-mutF and CR-*ler-*mutR. (ii) The tetRA cassette was removed with the same protocol using primer CR-*ler*-rmvl. Tetracycline sensitive clones were selected by growth on Tetracycline counterselective plates [35,36] at 32°C, for 2 days, yielding HA539 (Δ*ler*). (iii) Following the same protocol as (i), primers CR-*croI*-mutF and CR-*croI*-mutR were used to amplify the tetRA amplicon. The resulting amplicon was used to delete *croI* in *C*. *rodentium* resulting in strain HA528 (*croI*::*tetRA)*. A tetracycline-resistant strain of *C*. *rodentium* was constructed by inserting the tetRA cassette into the locus of *dadX*, using the primers CR-*dadX*-mut-F and CR-*dadX*-mut-R, yielding in strain HA538 (Δ*dadx*::*tetRA*). Plasmid pHA500 was derived from pM965 [39], by exchanging the ampicillin resistance with a kanamycin resistance cassette. This was done using pSIM9 and lambda red recombineering. A *kan* amplicon was amplified using pKD4 [40] as a template with primers pM965-kan-F and pM965-kan-R. The recombineering was carried out in an *E*. *coli* K12 strain. Plasmid pHA501 was derived from pM2120 [41], by exchanging the ampicillin resistance with a kanamycin resistance. This was done using pSIM9 and lambda red recombineering. A *kan* amplicon was amplified using pKD4 [40] as a template with primers pM2120-kan-F and pM2120-kan-R. The recombineering was carried out in an *E*. *coli* K12 strain. All deletions were verified phenotypically and by control PCR (control primers specified in S2 Table). Plasmids (see S1 Table for a complete list) were introduced by electroporation following standard protocols.

### Fluorescence microscopy

The distal 2 cm of the colon were fixed in 4% PFA in PBS for 24 h, then transferred to a 20% sucrose solution for 24–48 h. The fixed distal colon was then cut into three equally large parts and embedded in OTC media, and flash frozen with liquid nitrogen. Sections of 7 μm thickness were cut and rinsed twice in PBS and once in PBS/2%BSA. Sections were stained with a PBS/2%BSA solution containing DAPI (Sigma-Aldrich, USA, 0.01 mg/mL final concentration) and Phalloidin-Atto 647N (Sigma-Aldrich, USA, 0.04 nmol/mL final concentration) for 30min. Sections were rinsed again twice in PBS and one time in PBS/2%BSA and mounted under Vectashield (Vector laboratories, USA). Images were acquired using a Zeiss LSM710 Laser scanning microscope. In experiment with *C*. *rodentium* without a fluorescent plasmid, the bacteria were stained with an anti-*E*. *coli* O152 antiserum. Cryosections were washed twice in PBS and once in PBS/2%BSA and stained with a primary antibody (Rabbit anti *E*. *coli* O152 Antibody, Abcam, 1:400) for 1h. After washing with PBS the sections were stained with a secondary antibody (Alexa Fluor 488 AffiniPure goat anti-rabbit IgG, LucernaChem, USA, 1:400), DAPI (Sigma-Aldrich, USA, 0.01 mg/mL final concentration), and Phalloidin-Atto 647N (Sigma-Aldrich, USA, 0.04 nmol/mL final concentration) for 1h. Sections were rinsed again twice in PBS and one time in PBS/2%BSA and mounted under Vectashield (Vector laboratories, USA). Images were acquired using a Zeiss LSM710 Laser scanning microscope.

### Quantitation of A/E microcolonies

Fluorescently labelled distal colon sections were used to quantify microcolonies (see section “fluorescent microscopy”). For each mouse, three different horizontal sections of the distal colon were analyzed. Profiles of microcolonies were counted manually, a microcolony being defined as a cluster of bacteria on the epithelial surface interspaced by either uninfected epithelium from the next microcolony or by a microcolony of a different color. In order to analyze the percentage of infected surface, the whole horizontal section was imaged as a tile scan using either a Zeiss LSM710 Laser scanning microscope or a Zeiss Observer standard fluorescence microscope. The image was then sliced to acquire the original individual images that composed the tile (approx. 100 images per horizontal section of colon). Sections containing parts of the epithelial surface were selected. Of this selection every fifth image was chosen for analysis, choosing the first picture to be analyzed randomly (random number between 1 and 5 was drawn). This selection yielded in approx. 20 images per mouse, originating from all three original sections. Images were then analyzed using the STEPanalyzer stereology tool [18]. As horizontal sections were chosen, and no vertical design was applied, the stereological analysis is only valid in relative numbers, not absolute.

### Histopathology scoring

The same colon tissue as used for fluorescent imaging was processed for histopathology evaluation. The distal 2 cm of the colon were fixed in 4% PFA in PBS for 24 h, then transferred to a 20% sucrose solution for 24–48 h. The fixed distal colon was then cut into three equally large parts and embedded in OTC media, and flash frozen with liquid nitrogen. Sections of 7 μm thickness were cut and stained with hematoxylin and eosin. Sections were scored by an expert pathologist in a blinded manner: Epithelial hyperplasia (scored based on percentage above the height of the control, where 0 = no change (up to 0.2 mm); 1 = 1–50%; 2 = 51–100%; 3 = > 100%); epithelial integrity (0 = no pathological changes detectable, 1 = epithelial desquamation, 2 = erosion of the epithelial surface (gaps of 1 to 10 epithelial cells/lesion), 3 = epithelial ulceration (gaps of > 10 epithelial cells/ lesion); inflammatory cell infiltrate (0 = <5 polymorphonuclear granulocytes (PMN)/ high-power field, 1 = 5 to 20 PMN/high-power field, 2 = 21 to 60 PMN/high-power field, 3 = > 60 PMN/high-power field); loss of goblet cells (0 = >28 goblet cells/high-power field, 1 = 11–28 goblet cells/high-power field, 2 = 1 to 10 goblet cells/high-power field, 3 = < 1 goblet cells/high-power field); and submucosal edema (0 = no pathological changes, 1 = mild edema, 2 = moderate edema, 3 = profound edema); The reported combined score of maximal 15 is the sum of the individual scores. This scoring system was adapted from references [42] and [43].

### Lipocalin-2 ELISA

Fecal lipocalin-2 was measured using the solid phase Sandwich ELISA Mouse Lipocalin-2/NGAL DuoSet (R&D Systems, USA). The assay was performed according to the manufacturer’s instructions, except for the horseradish peroxidase, HRP-SA which was used from Biolegend (USA). Fecal pellets from mice were dissolved in 0.5 mL sterile PBS and debris removed by centrifugation (microfuge 7000 rpm, 5min). The supernatant was serially diluted in triplicates (1:3 dilution steps, 6 or 12 dilutions). The standard was serially diluted in duplicates (1:3 dilution steps, 12 dilutions). The OD_405_ was measured using a Thermomax microplate reader (Molecular Devices, USA) Data was analyzed using graph pad Prism program. A four parameter dose-response curve was fitted, using equal hill slopes for all samples on the same plate. Samples with low OD_405_ values resulting in a curve fit with R^2^ values below 0.95 were defined below detection limit. By comparison with the standard, the EC50 was used to calculate the absolute amount of Lipocalin-2 per sample.

### Bacterial RT-qPCR

For validation of the method on *in-vitro* grown bacteria (S2C Fig), an overnight culture was prepared in LB and grown for 16 h at 37°C, 150 rpm. The culture was then diluted 1:100 in either LB and grown at 37°C, 150 rpm or in DMEM (Gibco, Netherlands) and grown at 37°C, 5% CO_2_ without shaking. To determine density, at the time of sampling the OD_600_ was determined. Samples were removed at different time points, volumes adjusted to yield approx. 1x10^9^ bacteria (OD_600_ = 1). Samples were spun down (4500 g, 5 min), and resuspended in RNAprotect reagent (Qiagen, Germany). After 5 min incubation the bacterial suspension was spun again and the pellet resuspended in 200 μL lysis buffer (TE buffer containing 10 mg/mL Lysozyme, Sigma-Aldrich, Canada). The bacteria were incubated at room temperature for 10 min. RNA was isolated using the RNeasy mini kit (Qiagen, Germany), including the on column DNase digestion step. Concentration of RNA was measured using Nanodrop (ThermoScientific). Quality of the samples was tested with the RNA 6000 Nano kit (Agilent, USA). Colon contents were collected from mice and suspended in 5 mL sterile PBS. Large debris were sedimented for 10 sec and the supernatant transferred to a new tube. This solution containing the bacteria was spun down (4700 g, 7 min) and the pellet resuspended in 0.5 mL RNAprotect reagent prior to RNA isolation. Quantitative PCR (qPCR) was performed in duplicates or triplicates in a volume of 20 μL containing 600 ng RNA using the TaqMan RNA-to-CT 1-Step kit (Applied Biosystems, USA) and the Quant Studio 7 Flex System (ThermoFisher, USA). Following primers and probes were used: Ler-F-primer: GAG CAG GAG ATT CAA ACT GTA A; Ler-R-primer: CGT CTT CAT TAC GGT AGT ATA CC; ler-probe: CGG CGA GCA AGA GCA CCA TCA (modifications: 6-carboxyfluorescein (FAM) and Black hole quencher (BHQ-1)); rpoD-F-primer: AAG CGA AAG TCC TGC GTA TG, rpoD-R-primer: GCT TCG ATC TGA CGG ATA CG rpoD-probe: CGA TAT GAA CAC CGA CCA CAC GCT G (modifications: Yakima yellow (YYE) and BHQ-1). All probes and primers were synthesized and tested by Microsynth (Switzerland). The following program was run: 15 min 48°C, 10 min 95°C followed by 50 cycles of 15 s 95°C and 1 min 60°C. Fluorescence was measured for each cycle. C_t_ values for both genes were averaged and the mean C_t_ of ler was subtracted from the mean C_t_ value of rpoD to normalize.

### Gene expression analysis of distal colon tissue

Colon tissue was emptied from all fecal matter, washed in sterile PBS and immediately stored in RNAlater (Qiagen, Germany). RNA was extracted from the tissue using the RNeasy kit (Qiagen, Germany) according to the manufacturer’s instructions. RNA quality was verified with the Agilent Bioanalyzer system using RNA micro chips (Agilent Technologies, USA). Using 400 ng RNA, cDNA was prepared using the RT2 Easy First Strand Kit (Qiagen) following manufacture`s protocol.

To measure expression level of 12 genes (Reg3g, S100a8, Cxcl9, Ncr1 (NKp46), Cxcl1, Ifng, Cx3cl1, Il17a, Ccl22, Nos2 (iNOS), Bcl6, Foxp3) a custom made plate was used (Qiagen). RT^2^ SYBR Green Master Mix (87.5 μL, 2x, Qiagen), cDNA (13.7 μL) and nuclease-free water (73.8 μL, Qiagen) were mixed and 10 μl added to each well. Plates were run on QuantStudio 7 Flex Real-Time PCR System (ThermoFisher, USA) according to manufacturer’s protocol. Fluorescence was measured for each cycle. Ct values for each gene were subtracted from the mean Ct value of the housekeeping genes to normalize (*Actb*). The upper CT limit was fixed to 35 cycles.

### Flow cytometry

Lamina propria lymphocytes: Cecum and colon was removed, longitudinally opened and incubated in HBSS medium supplemented with DTT (308 mg/L), EDTA (0.0005 M), glucose (1 g/L), horse serum (2%), HEPES (0.01 M), and NaHCO_3_ (2.1 g/L) at 37°C for 15 min on a shaker for 2–3 rounds. Tissue was washed twice with PBS and digested in 0.5 mg/mL collagenase at 37°C on a shaker for 40 min. Cells were filtered and loaded onto a 80%-40% Percoll-gradient. Interphase was collected and spun down (8 min, 800 X g, 4°C). Cells were resuspended in 300 μL PBS/ 2%BSA and Fc-blocked (Anti CD16/32 AB, clone 93, Biolegend) for 10 min. Staining with antibodies (Ly6G (FITC labeled, clone 1A8), MHCII (PE labeled, clone M5/114.15.2), CD45 (PerCP labeled, clone 30-F11), CD11b (Pac. Blue labeled, clone M1/70), Ly6C (Alexa Fluor 700 labeled, clone HK1.4), CD64 (BV711 labeled, clone X54-5/7.1) all from Biolegend) was performed for 1 h on ice in a volume of 100 μL. Cells were washed and fixed with fixation buffer (Lysing Solution BDBioscience) for 10 min at RT. Cells were washed and resuspended for acquisition on a SORP LSRII flow cytometer (Becton Dickinson). Data was analyzed using FlowJo software (Treestar, USA). Population frequencies were calculated as percentage of CD45^+^ population. The gating strategy is shown in S4 Fig.

Blood was collected from the vena cava using a 18G syringe precoated with 7.5% EDTA (Sigma). Blood (40 μL) was distributed in 96-well U-bottom shaped microplates. Cells were counted with a VetABC animal blood counter (Medical Solution) and stained in 20 μl antibody mix diluted in FACS buffer directly added to blood for 1h at 4°C (Ly6G (FITC labeled, clone 1A8), CD45 (PerCP labeled, clone 30-F11), CD115 (APC labeled, clone AFS98), CD11b (Pac. Blue labeled, clone M1/70), Ly6C (Alexa Fluor 700 labeled, clone HK1.4) all from Biolegend). After washing two times, blood cell lysis was performed using FACS Lysing Solution (BDBioscience) for 10 min at room temperature. Cells were washed and acquired on a SORP LSRII flow cytometer (Becton Dickinson). Data was analyzed using FlowJo software (Treestar, USA). The gating strategy is shown in S5 Fig.

### Cytokine quantifications

Distal colon tissue was washed in cold PBS and directly frozen in 500 μL of PBS containing 0.5% BSA, 0.5% Tergitol and protease inhibitor (Sigma-Aldrich). Tissue was lysed using a steel bead and a Qiagen tissue lyser (10 min, full speed) and centrifuged (20 min, 5000 g, 4°C). Supernatant was used undiluted for bioplex assay (Biorad). Concentration of the following cytokines was analyzed using the standard manufacturer’s protocol: GM-CSF, KC, IFN-g, IL-1b, IL-6, IL-10, IL-7a, IL-22, IL-18, IL-23p19, IL-33, MCP-1 (CCL2), and MIG (CXCL9). Samples were acquired using the Bio-Plex 3D instrument (Biorad) and the xPONENT Software (Luminex). Data was analyzed using the Bioplex Manager 6.1 (Biorad).

### Statistical tests

Statistical analysis was performed using the Prism software (GraphPad, LaJolla, CA, USA). Details for statistical tests used are given in the figure legends.

## Supporting information

S1 Table Strains and plasmids used in this study. (DOCX)

S2 Table Primers used in this study. (DOCX)

S1 Fig Dose titration of *C*. *rodentium* infection in germ-free mice. (**A**) Intestinal colonization (CFU/g feces) of *C*. *rodentium* following intestinal inoculation of germ-free mice with 10^1^ (diamonds), 10^2^ (triangles), 10^4^ (circles), 10^6^ (inverted triangles), 10^8^ (asterisks), and 10^10^ (squares) CFU of *C*. *rodentium*. Mice were inoculated with a 1:1 mixture of GFP and mCherry expressing *C*. *rodentium*, respectively. Horizontal dotted line indicates detection limit. (**B**) Quantitation of colonic microcolonies 7 days post infection in the animals shown in A. n = 3–5 per group. Data-points from 10^10^ CFU group are reproduced from the dataset shown in main Fig 1J for comparison. (**C**) Computational image analysis-based quantification of percentage of surface affected by A/E infection in the microscopy specimens analyzed in Fig 1J. Three colonic cross sections per individual were analyzed. (**D**) Correlation analysis of microcolonies and percentage of infected surface area data between d3 and d7 post-infection. ****, p < 0.0001 (Student`s t-test); F-test, Fisher’s test.
(TIF)

S2 Fig Analysis of *ler* expression and CroIR quorum sensing system. (**A**) Luminal colonization of germ-free mice infected for 16 h with 10^4^ (blue dots) or 10^10^ (red squares) CFU *C*. *rodentium* (n = 6 per group). Horizontal dotted line indicates detection limit. (**B**) RT-qPCR quantitation of *ler* transcription in colon content of the mice shown in A. Symbols represent individual mice. Expression of *ler* was normalized to expression of the housekeeping gene *rpoD*. (**C**) Validation of *ler* mRNA specific RT-qPCR on *in vitro* grown *C*. *rodentium* and verification of *croI* independency of *ler* expression. *C*. *rodentium* (black circles) or *C*. *rodentium* Δ*croI* (green triangles) was grown either in LB (filled symbols) or in DMEM (open symbols), and samples were removed at different time points during growth. Data shown are representative of three independent experiments. (**D**) Germ-free mice were infected with different doses of either *C*. *rodentium* wild type (black circles) or *C*. *rodentium* Δ*croI* (green triangles) and microcolonies were analyzed in distal colon after 3 days. (n = 5–7, pooled from 3 independent experiments) Dotted line represents OD_600_ at which *ler* expression decreased in all experiments performed. ns, statistically not significant; **, P < 0.01; ***, p < 0.001; ****, p < 0.0001 Student`s t-test (D) or one way ANOVA with Dunnett post-test (A).
(TIF)

S3 Fig High-dose related hypovirulence in Caspase1/11^-/-^ mice. (**A**) Luminal colonization and (**B**) numbers of A/E microcolonies in the distal colon of germ-free Caspase1/11^-/-^ mice infected with either 10^4^ (open red squares) or 10^10^ (open blue dots) CFU of *C*. *rodentium* for 3 or 7 days. Germ free wild type control mice infected with 10^10^ CFU *C*. *rodentium* for 7 days are represented by filled red squares. N = 2–4 per group, pooled from two independent experiments. (**C-F**) Representative fluorescent microscopy images of distal colon of germ-free Caspase1/11^-/-^ mice infected with 10^4^ CFU (C, E) and 10^4^ CFU (D, F) of *C*. *rodentium* analyzed on day 3 (C, D) and day 7 (E, F) post-infection. Every individual was infected with a 1:1 mixture of GFP and mCherry expressing bacteria. Grey, F-actin/phalloidin; green, GFP-expressing *C*. *rodentium*; red, mCherry-expressing *C*. *rodentium*. Insets indicate areas shown in higher-magnification panels. Scale bars: 200 μm (overview) or 100 μm (higher-magnification panels). Error bars indicate standard deviations. Dotted line marks lower detection limit. **, p < 0.01; statistical tests: Student`s t-test (panel B, day 3), 1-way ANOVA with Tukey posttest (panel B, day 7).
(TIF)

S4 Fig Colonic histology at 18 hours after infection. Representative H&E-stained histological sections of colonic tissue from germ-free mice infected for 18 hours with 10^4^ CFU of wild-type *C*. *rodentium* (**A**), 10^4^ CFU of avirulent *C*. *rodentium* Δ*ler* (**B**), and uninfected controls (**C**) reveal no overt signs of histopathology. Examples are representative for all individuals (n = 5) of each experimental group. L, intestinal lumen; Cr, crypt; Ep, Epithelium; M, muscularis mucosae; Scale bar: 100 μm. (**D**) Histopathological scores of n = 5 mice per experimental group; bar indicates mean; ns, statistically non-significant; statistical test: Kruskall-Wallis.
(TIF)

S5 Fig Flow cytometry gating strategy for peripheral blood and caecum/colon. (**A**) The leukocyte population in peripheral blood was defined by gating on single cells and further on CD45^+^ cells. Neutrophils were then defined as Ly6G^+^ and monocytes as CD11b^+^ and CD115^+^ cells. (**B**) The leukocyte population in caecum and colon was defined by gating on single cells and further on CD45^+^ cells. Neutrophils were then defined as CD11b^+^ Ly6G^+^ cells. The leukocyte population was further analyzed by gating on CD11b^+^ CD64^-^ cells (immature myeloid cells), exclusion of neutrophils (Ly6G^-^) and separation of infiltrating monocytes (Ly6C^high^ MHCII^-^) from resident monocytes.
(TIF)
