# Supplementary material for: Innate immunity restricts Citrobacter rodentium A/E pathogenesis initiation to an early window of opportunity
Source: PLoS Pathog. 2017 Jun 29;13(6):e1006476. doi: 10.1371/journal.ppat.1006476 (PMC5507559; doi:10.1371/journal.ppat.1006476)
Supplement: S1 Table — (DOCX) [file ppat.1006476.s001.docx]

| **Strain/Plasmid** | **Relevant genotype/phenotypes/description** | **Source/Reference** |
| --- | --- | --- |
| ***C. rodentium* ATCC51459** | Wild type | ATCC, Manassas VA |
| **HA526** | Δ*ler::tetRA*, Tet^R^ | This study |
| **HA539** | Δ*ler* | This study |
| **HA528** | Δ*croI::tetRA*, Tet^R^ | This study |
| **HA532** | Δ*croR::tetRA*, Tet^R^ | This study |
| **HA538** | Δ*dadX::tetRA*, Tet^R^ | This study |
| **pHA500** | Constitutive GFP-expression plasmid, Kan^R^ | This study |
| **pHA501** | Constitutive mCherry-expression plasmid, Kan^R^ | This study |
| **pSIM5** | Lambda-red expression plasmid, Cam^R^ | [37] |
| **pSIM9** | Lambda-red expression plasmid, Cam^R^ | [37] |
