# Supplementary material for: Innate immunity restricts Citrobacter rodentium A/E pathogenesis initiation to an early window of opportunity
Source: PLoS Pathog. 2017 Jun 29;13(6):e1006476. doi: 10.1371/journal.ppat.1006476 (PMC5507559; doi:10.1371/journal.ppat.1006476)
Supplement: S2 Table — (DOCX) [file ppat.1006476.s002.docx]

| **Name** | **Description** | **Sequence** |
| --- | --- | --- |
| **CR-*ler*-mutF** | *ler tetRA* cassette construction forward | TCCAGTTCAGTTATCGTTATCATTTAATTATTTCATGTTACTAAGCACTTGTCTCCTG |
| **CR-*ler*-mutR** | *ler tetRA* cassette construction reverse | ATGTAAGGATGAGCTTGTTAATATCTTAATATATAAAAGTTTAAGACCCACTTTCACATT |
| **CR-*ler*-rmvl** | *ler tetRA* removal primer | TCCAGTTCAGTTATCGTTATCATTTAATTATTTCATGTTAACTTTTATATATTAAGATATTAACAAGCTCATCCTTACAT |
| **CR-*ler*-cntrF** | *ler* control primer forward | CCTTAATTGCCGCATCGCAT |
| **CR-*ler*-cntrR** | *ler* control primer reverse | GGCGAGCCGCTTACTCTAAA |
| **CR-*croI*-mutF** | *croI tetRA* cassette construction forward | GCTCAGATTACTGGATAACTGAGCCCCGGATACCTGCTTACTAAGCACTTGTCTCCTG |
| **CR-*croI*-mutR** | *croI tetRA* cassette construction reverse | AGGGGATAATAATTTCAGTGTGTGATTATCAGGAATAACATTAAGACCCACTTTCACATT |
| **CR-*croR*-mutF** | *croR tetRA* cassette construction forward | TATACATCTTCAACCATTCATCCAAGAGGCTGTAACTGACTTAAGACCCACTTTCACATT |
| **CR-*croR*-mutR** | *croR tetRA* cassette construction reverse | TCAACAACTGCGCCGTATGTAAGCAGGTATCCGGGGCTCACTAAGCACTTGTCTCCTG |
| **CR-*cro*-cntrF** | *cro* control primer forward | AAAGATCCCGACCACCGAAC |
| **CR-*cro*-cntrR** | *cro* control primer reverse | AAAACCCAAGGGGCAACAGA |
| **CR-*dadX*-mutF** | *dadX tetRA* cassette construction forward | CGGTCAACTGCACGGCGCACATAACTAAGAAGGACGCGAGTTAAGACCCACTTTCACATT |
| **CR-*dadX*-mutR** | *dadX tetRA* cassette construction reverse | ATAAGGCGCATCGCGCCATCCGGCAATAAAGGAAAAGTTACTAAGCACTTGTCTCCTG |
| **CR-*dadX*-cntrF** | *dadX* control primer forward | AAATTTGCCGATGTGGCGTT |
| **CR-*dadX*-cntrR** | *dadX* control primer reverse | CTTTCTGGACCGGGTTACGG |
| **pM965-kan-F** | pM965 *kan* cassette construction forward | AATCAATCTAAAGTATATATGAGTAAACTTGGTCTGACAGCATATGAATATCCTCCTTA |
| **pM965-kan-R** | pM965 *kan* cassette construction reverse | ACCCTGATAAATGCTTCAATAATATTGAAAAAGGAAGAGTGTGTAGGCTGGAGCTGCTTC |
| **pM965-kan-cntrF** | pM965 control primer forward | CGCTCAGTGGAACGAAAACTC |
| **pM965-kan-cntrR** | pM965 control primer reverse | CGCGGAACCCCTATTTGTTT |
| **pM2120-kan-F** | pM2120 *kan* cassette construction forward | TGCGTTTCTACAAACTCTTTTGTTTATTTTTCTAAATACAGTGTAGGCTGGAGCTGCTTC |
| **pM2120-kan-R** | pM2120 *kan* cassette construction reverse | AATCAATCTAAAGTATATATGAGTAAACTTGGTCTGACAGCATATGAATATCCTCCTTA |
| **pM2120-kan-cntrF** | pM2120 control primer forward | CATCCTGACGGATGGCCTTT |
| **pM2120-kan-cntrR** | pM2120 control primer reverse | GCTACAGGGCGCGTAAATCA |
