# Supplementary figures and images for: Innate immunity restricts Citrobacter rodentium A/E pathogenesis initiation to an early window of opportunity
Source: PLoS Pathog. 2017 Jun 29;13(6):e1006476. doi: 10.1371/journal.ppat.1006476 (PMC5507559; doi:10.1371/journal.ppat.1006476)

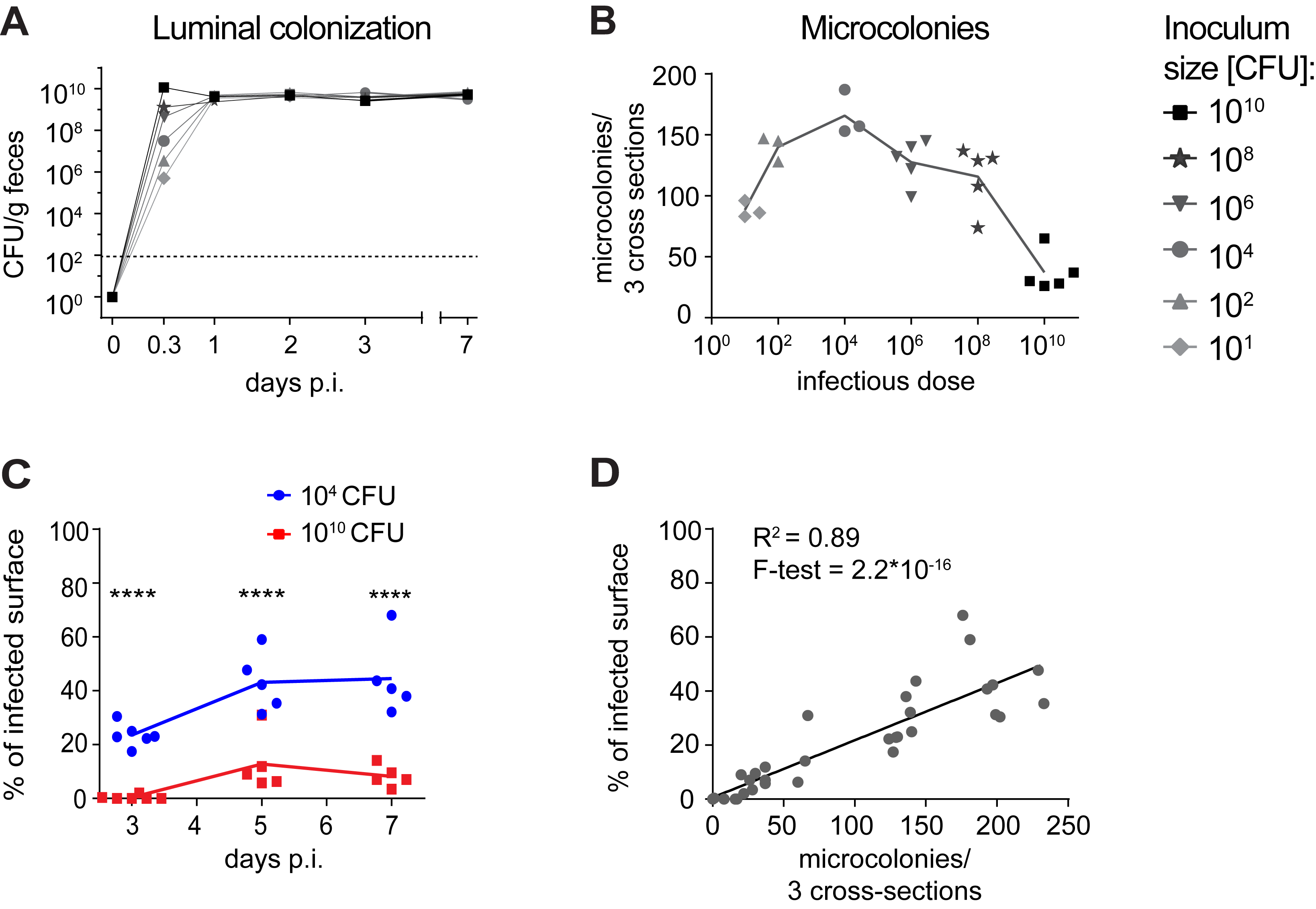

Supplement: S1 Fig — (A) Intestinal colonization (CFU/g feces) of C. rodentium following intestinal inoculation of germ-free mice with 101 (diamonds), 102 (triangles), 104 (circles), 106 (inverted triangles), 108 (asterisks), and 1010 (squares) CFU of C. rodentium. Mice were inoculated with a 1:1 mixture of GFP and mCherry expressing C. rodentium, respectively. Horizontal dotted line indicates detection limit. (B) Quantitation of colonic microcolonies 7 days post infection in the animals shown in A. n = 3–5 per group. Data-points from 1010 CFU group are reproduced from the dataset shown in main Fig 1J for comparison. (C) Computational image analysis-based quantification of percentage of surface affected by A/E infection in the microscopy specimens analyzed in Fig 1J. Three colonic cross sections per individual were analyzed. (D) Correlation analysis of microcolonies and percentage of infected surface area data between d3 and d7 post-infection. ****, p < 0.0001 (Student`s t-test); F-test, Fisher’s test. (TIF) [file ppat.1006476.s003.tif]

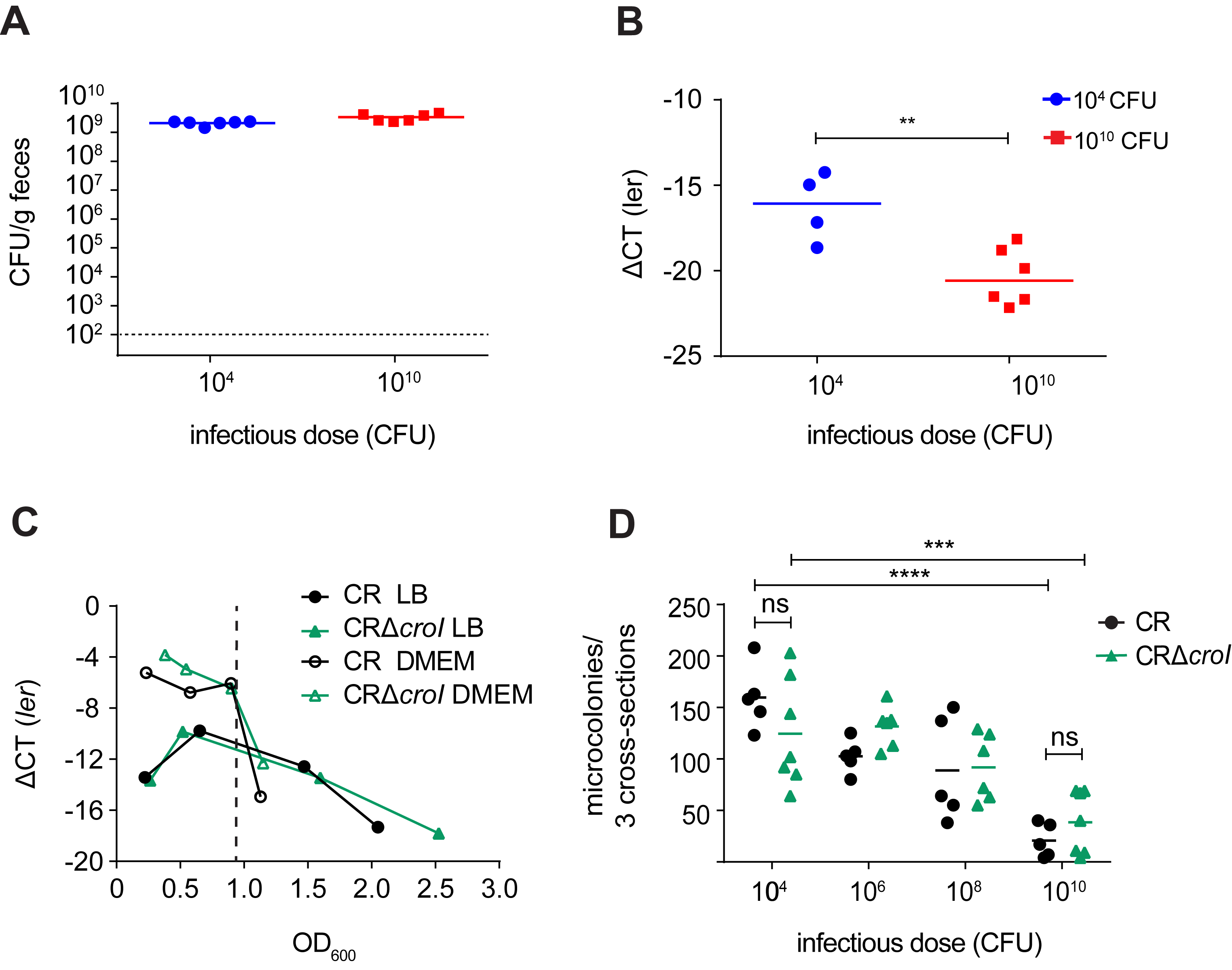

Supplement: S2 Fig — (A) Luminal colonization of germ-free mice infected for 16 h with 104 (blue dots) or 1010 (red squares) CFU C. rodentium (n = 6 per group). Horizontal dotted line indicates detection limit. (B) RT-qPCR quantitation of ler transcription in colon content of the mice shown in A. Symbols represent individual mice. Expression of ler was normalized to expression of the housekeeping gene rpoD. (C) Validation of ler mRNA specific RT-qPCR on in vitro grown C. rodentium and verification of croI independency of ler expression. C. rodentium (black circles) or C. rodentium ΔcroI (green triangles) was grown either in LB (filled symbols) or in DMEM (open symbols), and samples were removed at different time points during growth. Data shown are representative of three independent experiments. (D) Germ-free mice were infected with different doses of either C. rodentium wild type (black circles) or C. rodentium ΔcroI (green triangles) and microcolonies were analyzed in distal colon after 3 days. (n = 5–7, pooled from 3 independent experiments) Dotted line represents OD600 at which ler expression decreased in all experiments performed. ns, statistically not significant; **, P < 0.01; ***, p < 0.001; ****, p < 0.0001 Student`s t-test (D) or one way ANOVA with Dunnett post-test (A). (TIF) [file ppat.1006476.s004.tif]

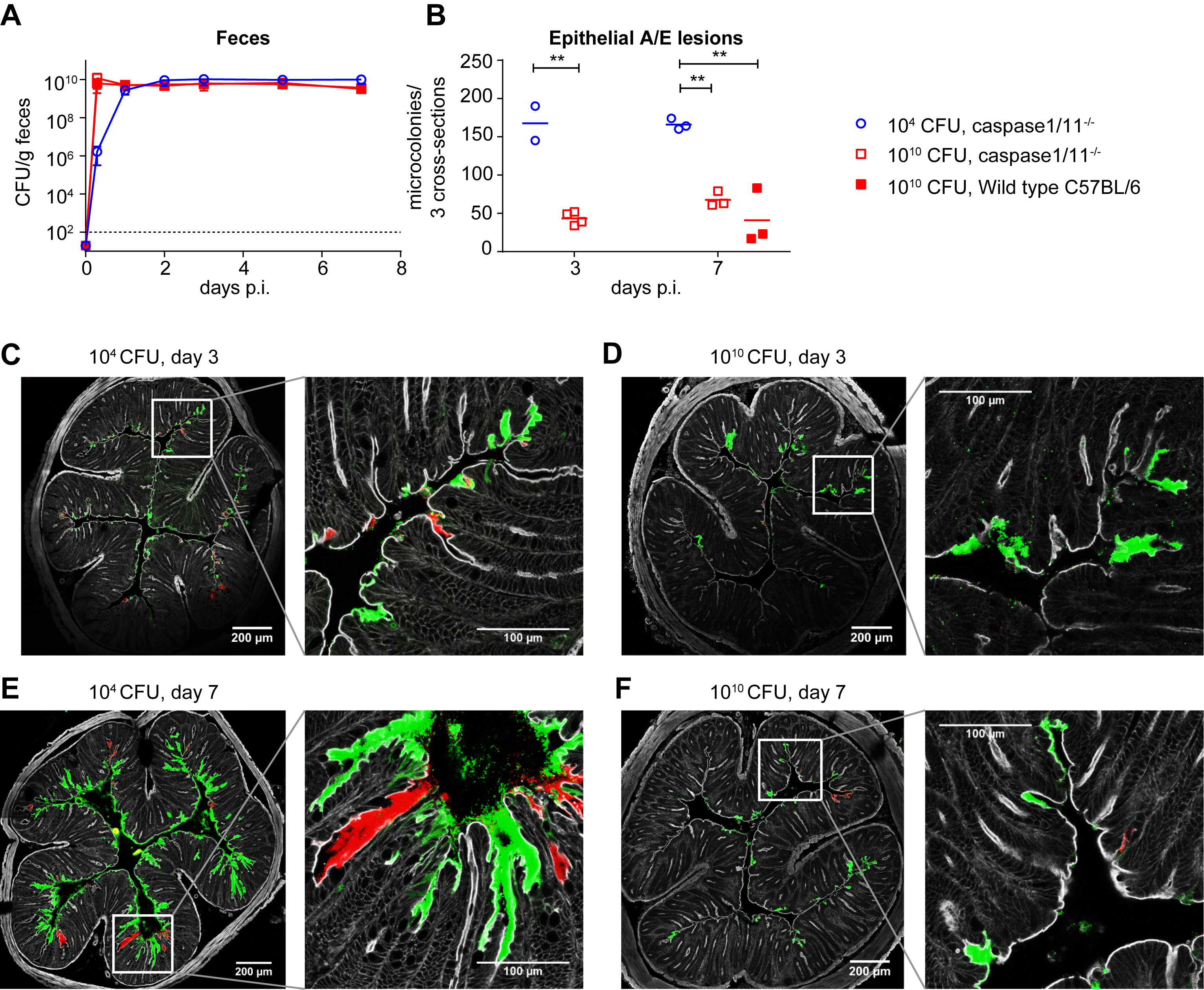

Supplement: S3 Fig — (A) Luminal colonization and (B) numbers of A/E microcolonies in the distal colon of germ-free Caspase1/11-/- mice infected with either 104 (open red squares) or 1010 (open blue dots) CFU of C. rodentium for 3 or 7 days. Germ free wild type control mice infected with 1010 CFU C. rodentium for 7 days are represented by filled red squares. N = 2–4 per group, pooled from two independent experiments. (C-F) Representative fluorescent microscopy images of distal colon of germ-free Caspase1/11-/- mice infected with 104 CFU (C, E) and 104 CFU (D, F) of C. rodentium analyzed on day 3 (C, D) and day 7 (E, F) post-infection. Every individual was infected with a 1:1 mixture of GFP and mCherry expressing bacteria. Grey, F-actin/phalloidin; green, GFP-expressing C. rodentium; red, mCherry-expressing C. rodentium. Insets indicate areas shown in higher-magnification panels. Scale bars: 200 μm (overview) or 100 μm (higher-magnification panels). Error bars indicate standard deviations. Dotted line marks lower detection limit. **, p < 0.01; statistical tests: Student`s t-test (panel B, day 3), 1-way ANOVA with Tukey posttest (panel B, day 7). (TIF) [file ppat.1006476.s005.tif]

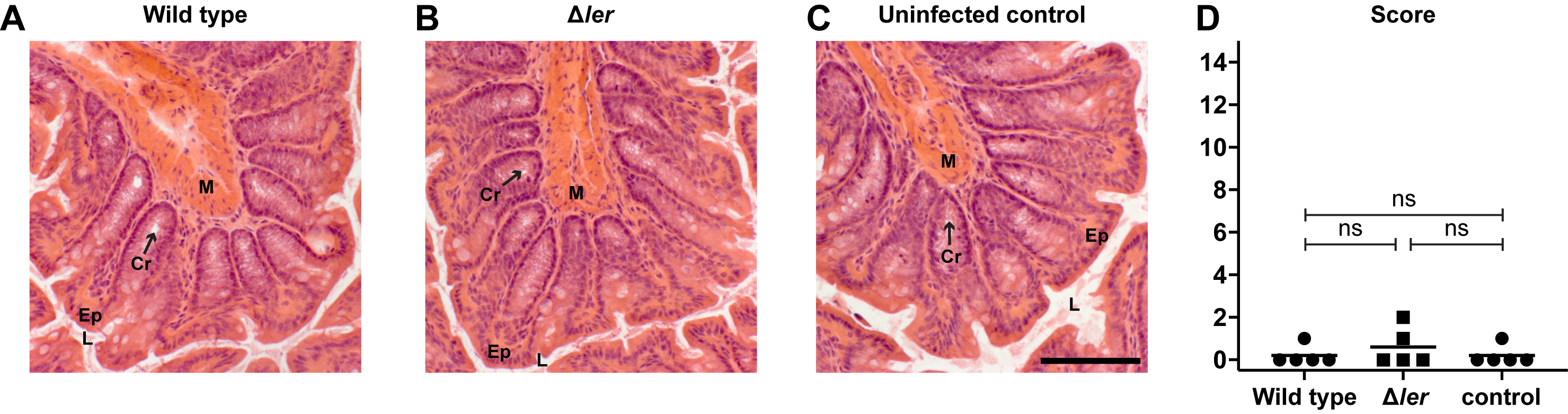

Supplement: S4 Fig — Representative H&E-stained histological sections of colonic tissue from germ-free mice infected for 18 hours with 104 CFU of wild-type C. rodentium (A), 104 CFU of avirulent C. rodentium Δler (B), and uninfected controls (C) reveal no overt signs of histopathology. Examples are representative for all individuals (n = 5) of each experimental group. L, intestinal lumen; Cr, crypt; Ep, Epithelium; M, muscularis mucosae; Scale bar: 100 μm. (D) Histopathological scores of n = 5 mice per experimental group; bar indicates mean; ns, statistically non-significant; statistical test: Kruskall-Wallis. (TIF) [file ppat.1006476.s006.tif]

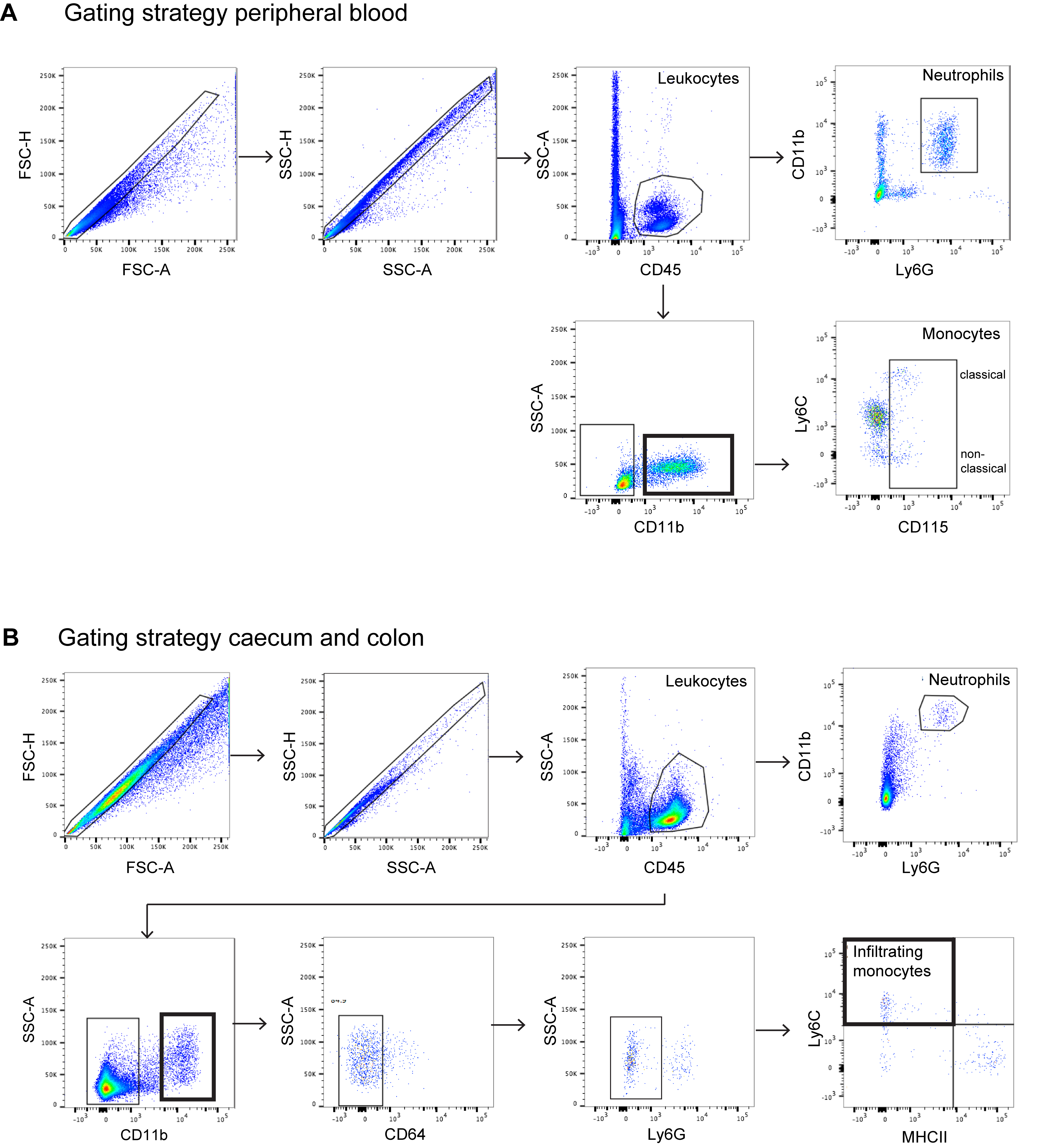

Supplement: S5 Fig — (A) The leukocyte population in peripheral blood was defined by gating on single cells and further on CD45+ cells. Neutrophils were then defined as Ly6G+ and monocytes as CD11b+ and CD115+ cells. (B) The leukocyte population in caecum and colon was defined by gating on single cells and further on CD45+ cells. Neutrophils were then defined as CD11b+ Ly6G+ cells. The leukocyte population was further analyzed by gating on CD11b+ CD64- cells (immature myeloid cells), exclusion of neutrophils (Ly6G-) and separation of infiltrating monocytes (Ly6Chigh MHCII-) from resident monocytes. (TIF) [file ppat.1006476.s007.tif]
